# Effectiveness of Cash Transfer Delivered Along With Combination HIV Prevention Interventions in Reducing the Risky Sexual Behavior of Adolescent Girls and Young Women in Tanzania: Cluster Randomized Controlled Trial

**DOI:** 10.2196/30372

**Published:** 2022-09-19

**Authors:** Evodius Kuringe, Alice Christensen, Jacqueline Materu, Mary Drake, Esther Majani, Caterina Casalini, Deusdedit Mjungu, Gaspar Mbita, Esther Kalage, Albert Komba, Daniel Nyato, Soori Nnko, Amani Shao, John Changalucha, Mwita Wambura

**Affiliations:** 1 Department of Sexual and Reproductive Health National Institute for Medical Research Mwanza United Republic of Tanzania; 2 Sauti Project Jhpiego (an affiliate of John Hopkins University) Dar-es-Salaam United Republic of Tanzania

**Keywords:** adolescent, female, HIV infections/epidemiology, HIV infections/prevention and control, herpes simplex virus type 2, incidence, motivation, Tanzania

## Abstract

**Background:**

Poverty and social inequality exacerbate HIV risk among adolescent girls and young women (AGYW) in sub-Saharan Africa. Cash transfers can influence the structural determinants of health, thereby reducing HIV risk.

**Objective:**

This study assessed the effectiveness of cash transfer delivered along with combination HIV prevention (CHP) interventions in reducing the risky sexual behavior of AGYW in Tanzania. The incidence of herpes simplex virus type 2 (HSV-2) infection was used as a proxy for sexual risk behavior.

**Methods:**

A cluster randomized controlled trial was conducted in 15 matched pairs of communities (1:1 intervention to control) across 3 strata (urban, rural high-risk, and rural low-risk populations) of the Shinyanga Region, Tanzania. The target population was out-of-school AGYW aged 15-23 years who had completed 10-hour sessions of social and behavior change communication. Eligible communities were randomly assigned to receive CHP along with cash transfer quarterly (intervention group) or solely CHP interventions (control group) with no masking. Study recruitment and baseline survey were conducted between October 30, 2017 and December 1, 2017. Participants completed an audio computer-assisted self-interview, HIV counselling and testing, and HSV-2 testing at baseline and during follow-up visits at 6, 12, and 18 months after the baseline survey. A Cox proportional hazards model with random effects specified at the level of clusters (shared frailty) adjusted for matching pairs and other baseline imbalances was fitted to assess the effects of cash transfer on the incidence of HSV-2 infection (primary outcome). Secondary outcomes included HIV prevalence at follow-up, self-reported intergenerational sex, and self-reported compensated sex. All secondary outcomes were measured at each study visit.

**Results:**

Of the 3026 AGYW enrolled in the trial (1482 in the intervention and 1544 in the control), 2720 AGYW (1373 in the intervention and 1347 in the control) were included in the final analysis. Overall, HSV-2 incidence was not significantly different at all follow-up points between the study arms in the adjusted analysis (hazard ratio 0.96, 95% CI 0.67-1.38; *P*=.83). However, HSV-2 incidence was significantly lower in the rural low-risk populations who received the cash transfer intervention (hazard ratio 0.45, 95% CI 0.29-0.71; *P*=.001), adjusted for potential confounders.

**Conclusions:**

Although this trial showed no significant impact of the cash transfer intervention on HSV-2 incidence among AGYW overall, the intervention significantly reduced HSV-2 incidence among AGYW in rural low-risk communities. Factors such as lesser poverty and more asset ownership in urban and rural high-risk communities may have undermined the impact of cash transfer.

**Trial Registration:**

ClinicalTrials.gov NCT03597243; https://clinicaltrials.gov/show/NCT03597243

## Introduction

Adolescent girls and young women (AGYW) aged 15-24 years in eastern and southern regions of Africa have high rates of HIV infection [1-4]. In Tanzania, the HIV incidence was 0.14% (95% CI 0%-0.31%) and 0% (95% CI 0%-0.23%) in 2016 among AGYW and adolescent boys and young men, respectively [5]. Targeted HIV prevention efforts among AGYW are crucial to realize an HIV-free generation [6]. Interactions among biological [7-9], behavioral [10-15], and structural determinants of health [10,16] have been associated with higher HIV infection risks in AGYW, with poverty [10,16] and gender inequality [10,17] exacerbating their vulnerability. AGYW from low-income households are likely to engage in transactional sex [12,15] to cater to their daily subsistence and are less likely to negotiate safer sexual practices [9,10,18]. This highlights the need for incorporating structural interventions in combination with biomedical and behavioral approaches for impactful HIV prevention among AGYW [19-21]. Cash transfer as a structural intervention has shown promising results, with some studies demonstrating its impact on the prevention of sexually transmitted infections and delaying marriage and childbearing [22-25]. Of the 8 cash transfer studies conducted in sub-Saharan Africa with sexually transmitted infections as an outcome, 4 reported a significant reduction; cash transfer provided to in-school AGYW aged 13-22 years and their parents reduced HIV prevalence by 64% (95% CI 9%-86%) and herpes simplex virus type 2 (HSV-2) prevalence by 76% (95% CI 35%-91%) [22]. Short-term financial incentives to engage in safe sex in Lesotho reduced HIV incidence among males and females aged 18-30 years by 25% (95% CI 3%-42%) [25]. Similarly, another financial incentive to engage in safe sex in Eswatini, which was conditional on staying sexually transmitted infection–negative, reduced HIV incidence among schoolgirls by 23% (95% CI 1%-40%) compared to those not eligible for educational cash transfer [23,26]. A South African study [27,28] showed reduced HSV-2 incidence by 30% (95% CI 14%-43%) among school-based girls and boys but had too few new infections for HIV incidence analysis. Other studies [29,30] reported that cash transfer had no effect on HIV prevalence in South Africa among in-school adolescent girls (adjusted odds ratio [OR] 1.17, 95% CI 0.80-1.72) [29] and among men and women in Malawi conditional on remaining HIV-negative (β=.001, robust SE=.005) [30]. Cash transfer studies among in-school adolescent orphans showed no effect on HIV infection (OR 1.15, 95% CI 0.47-2.79) and HSV-2 infection (OR 1.46, 95% CI 0.50-4.26) in Zimbabwe [31] and on HIV infection (adjusted OR 0.72, 95% CI 0.15-3.42) and HSV-2 infection (OR 0.98, 95% CI 0.54-4.26) in Kenya [32]. However, none of these studies in sub-Saharan Africa were conducted among out-of-school AGYW aged 15-23 years to ascertain the combined effect of cash transfer and combination HIV prevention (CHP) services.

We examined the synergetic effect of cash transfer and CHP interventions among out-of-school AGYW in the Shinyanga Region, Tanzania, where the Sauti (meaning *Voices in Kiswahili)* project provided CHP interventions to AGYW [33]. We had 2 aims for this study. First, we sought to assess the synergetic effect of cash transfer and CHP interventions on HSV-2 incidence. Second, we sought to examine the effect of cash transfer along with CHP on AGYW’s sexual behavior. We hypothesized that cash transfer along with CHP interventions would be associated with a reduced risky sexual behavior compared with CHP alone. HSV-2 incidence was chosen as a proxy measure for HIV incidence, as the study would not be powered to detect a difference in HIV incidence in a relatively low incidence setting like Tanzania. Other measures of risky sexual behavior such as self-reported behavioral indicators are affected by bias and low validity compared to biomarkers such as HIV and HSV-2 infection [34,35]. HSV-2 is a sexually transmitted infection like HIV, more prevalent in Tanzania, and has been used as a marker for risky sexual behavior in other similar studies conducted in sub-Saharan Africa [29,36]. Our findings contribute to the body of evidence on the effectiveness of cash transfer in the reduction of risky sexual behavior among AGYW in Tanzania.

## Methods

### Study Design and Setting

This study was a 2-arm cluster-randomized controlled trial with 1:1 allocation ratio, implemented among out-of-school AGYW in Tanzania by the Sauti project—a project implementing community-based interventions under the DREAMS (Determined, Resilient, Empowered, AIDS-free, Mentored, and Safe) initiative in Tanzania [4,33]. The Sauti project collaborated with the Government of Tanzania and civil society organizations (CSOs) to provide community-based CHP interventions to AGYW in selected regions of Tanzania, which is the largest country in East Africa and is hierarchically subdivided into regions, districts, divisions, wards, and villages in rural settings and into neighborhoods *(mtaa)* in urban settings. A description of the methods used in this trial has been previously published [33]. Briefly, this study used 15 matched clusters randomly allocated to intervention (cash transfer plus CHP) or control (CHP only) arms. We randomly assigned clusters rather than individuals because cash transfer could be shared among family members even when allocated in different arms (risk of contamination: AGYW are likely to share the cash given to the participant in the intervention arm with relatives allocated to the control arm, thus reducing/diluting the impact of the study) [37,38]. In addition, a 5-km buffer zone was maintained between clusters to minimize the dilution of the intervention.

### Ethics Approval

Ethics approval for this study was obtained from the Johns Hopkins University School of Public Health Research Ethics Committee (00007976) and the Tanzanian Medical Research Coordinating Committee of the National Institute for Medical Research (NIMR/HQ/R.8a/VolIX/2287). This trial was registered at ClinicalTrials.gov (NCT03597243) and was conducted and reported following the Consolidated Standards of Reporting Trials (CONSORT) guidelines for cluster randomized trials [39].

### Selection of Participants

This study enrolled participants from a pool of potential DREAMS beneficiaries in the Shinyanga Region. The project conducted a household survey among AGYW in the identified villages to determine AGYW who were out-of-school and developed a pool of potential cash transfer program beneficiaries. Potential beneficiaries were then invited to attend 10 sessions of social and behavior change communication (SBCC) and other project interventions. The SBCC sessions were peer-led group sessions designed to address significant determinants of HIV risk, gender, and reproductive health. During the SBCC sessions, data on all villages receiving CHP were generated for study eligibility assessment and randomization. In the identified study clusters (intervention and control), potential participants were also given information about the study during group meetings for SBCC. After 10 hours of SBCC sessions, AGYW were informed about study enrolment, recruitment dates, and locations. AGYW were eligible for the study if they were aged 15-23 years; out-of-school (defined as either never enrolled or have dropped out of school for at least a month at the time of study enrolment) as documented by the household survey; residents of the village of recruitment; had completed 10 hours of SBCC training; willing to take part in the study, including testing for HIV and receiving results; and willing to participate in the cash transfer program (applicable to the intervention arm). Each study participant provided written informed consent or assent. Consent and assent forms were administered in Kiswahili. AGYW who tested positive for HIV or HSV-2 infection at baseline were enrolled in the study to avoid inadvertent disclosure of their serostatus. However, those with a positive HSV-2 status at baseline were excluded from the main analysis.

### Randomization and Masking

Villages were eligible for the study if they were receiving other Sauti project interventions except for cash transfer, identified as potentially eligible for cash transfer program, and having between 110 and 150 AGYW aged 15-23 years who were out of school. All eligible clusters were matched into pairs by location (rural vs urban areas) and the presence or absence of HIV high-risk areas, generating 3 strata: rural clusters in the high-risk area, urban clusters, and rural clusters in the low-risk area. Matching was conducted to minimize the between-community variance in HSV-2 incidence within the matched clusters. One cluster was randomly selected from each matched pair to receive the intervention package, and the other was automatically assigned to the control arm. No blinding was performed in this study.

### Study Implementation

Participants in the intervention arm received CHP and unconditional cash transfer in quarterly instalments of 70,000 Tanzania shillings (~US $31) for 18 months through mobile money on a project-provided cellular phone. Participants in the control arm received CHP only and cellular phones provided by the project. The aim was to make sure that the 2 arms were comparable except for the intervention. The interventions are described elsewhere [33]. In short, all study participants received Sauti’s core package of interventions, including risk reduction counselling, HIV testing services, condom use skills and provision, family planning counselling and service provision, sexually transmitted infection screening and treatment, gender-based violence interventions (escorted referrals and the desk for social, legal, and medical services provided to survivors of gender-based violence), tuberculosis and alcohol and drug abuse screening, and referral to services. The other features were SBCC training sessions and economic empowerment community banking also called as the Women Organizing Resources Together plus (WORTH+) intervention [33]. The cash transfer program was implemented in the intervention arm only. The WORTH+ intervention consisted of financial literacy training that aimed to build microbusiness development skills and community banking.

At baseline, following consent and enrolment into the study, AGYW completed audio computer-assisted self-interview (ACASI), which collected data on demographic information, factors related to HIV vulnerabilities, family planning, sexual risk behavior, and gender-based violence. Sexual behavior data collected included compensated sex (sexual encounters motivated by exchange for money, material support, or other benefits) and intergenerational sex (a sexual partnership between AGYW and a man 10 or more years older). Further, data on sex work defined as having negotiated payment for sex and transactional sex defined as initiating a sexual relationship with an expectation to receive money or gifts were collected. After data collection using ACASI, trained government health care workers offered HIV pretest and posttest counselling to participants. Blood was drawn for HIV and HSV-2 testing. HIV testing was done following the National Guidelines for the Management of HIV and AIDS [40]. All study procedures were conducted in a confidential environment in preidentified venues in the respective communities. Participants were seen every 6 months for study activities (6, 12, and 18 months), while the Sauti program provided CHP interventions. Each study visit included ACASI, HIV pretest and posttest counselling, and HSV-2 testing (if negative at the previous visit). Blood specimens were taken and transported to the local health facility laboratory for serum separation and temporarily stored at –20 °C before transportation to the National Institute for Medical Research (Mwanza laboratory) for HSV-2 testing.

HIV testing was conducted using 2 HIV rapid tests following Tanzania’s national HIV testing and counselling guidelines. Participants who were HIV-positive received escorted referrals to care and treatment centers. HSV-2 testing was conducted using the HSV-2 IgG enzyme-linked immunosorbent assay (Kalon Biological Ltd). Participants with positive HSV-2 test results were given posttest counselling and referral for treatment where required. At each follow-up visit, nurse counsellors assessed the social harm events by actively interrogating the study participant. If social harm was reported, it was recorded and graded per Sauti project safety guidelines, which defined and outlined procedures for reporting and management. All social harms were reported to the study steering committee, and the Sauti project initiated investigations and responses as appropriate. It was anticipated that any harm to AGYW owing to study participation would be minimal. The primary outcome was HSV-2 incidence, while secondary outcomes included HIV prevalence at follow-up, self-reported intergenerational sex, and self-reported compensated sex. All secondary outcomes were measured at each study visit.

### Statistical Analysis

All calculations for sample size were conducted using methods for matched cluster-randomized trials [41]. A sample of 14 paired clusters (28 clusters) with 70 participants per cluster was estimated to achieve over 80% power of detecting a 35% reduction in HSV-2 incidence in the intervention arm at the end of 18 months. The within-pair coefficient of variation between clusters was assumed to be 0.25 [42], and the significance level of the test was .05. It was estimated that, at baseline, HSV-2 prevalence would be 20% among AGYW aged 15-23 years [43], the attrition rate would be 10%, and the nonresponse rate would be 18% over 18 months. Thus, the sample size (70 AGYW per cluster) was increased to 104 AGYW per cluster (1560 per arm) and paired clusters increased to 15 (30 clusters). The HSV-2 incidence estimate was based on a sample size of approximately 1575 person-years per cluster by month 18. All analyses performed were prespecified. The primary analysis was intention-to-treat and based on individual-level data because the study had a sufficient number of clusters per arm, and the cluster size was anticipated to differ considerably at follow-up [41].

Descriptive analysis was done using standard methods for the analysis of a pair-matched cluster randomized trial with a small number of clusters [44]. Baseline data were used to assess balance across study arms in sociodemographic and other key characteristics associated with an outcome. Since covariates such as age, marital status, whether AGYW had emotional/psychological support, whether AGWY had debt at the time of the survey, and whether AGYW lacked food in the past 4 weeks were imbalanced across the study arms, they were adjusted for in the analysis.

A Cox proportional hazards model with random effects specified at the level of clusters (shared frailty), adjusted for matching pairs and other baseline imbalances, was fitted to assess the effects of cash transfer on the incidence of HSV-2 infection. The significance of the intervention was assessed using a value of .05 (2-sided) after verification of the validity of the proportional hazards assumption. Secondary analysis of the effect of cash transfer on HIV prevalence at follow-up was estimated using a log-binomial model adjusting for matched pairs, age, and other variables with baseline imbalance between the arms and adjusting for standard errors for clustering at the village level. Intergenerational sex, compensated sex, transactional sex, and other behavioral outcomes were compared between study arms by using generalized estimating equations with identity logit, binomial distribution, and robust variance to account for repeated measures on each participant. The generalized estimating equations regression models were also fitted to assess for interaction between the trial arm and strata (rural cluster in the high-risk area, urban cluster, rural cluster in the low-risk area).

## Results

### Sociodemographic Characteristics of the Participants

**Figure 1:**
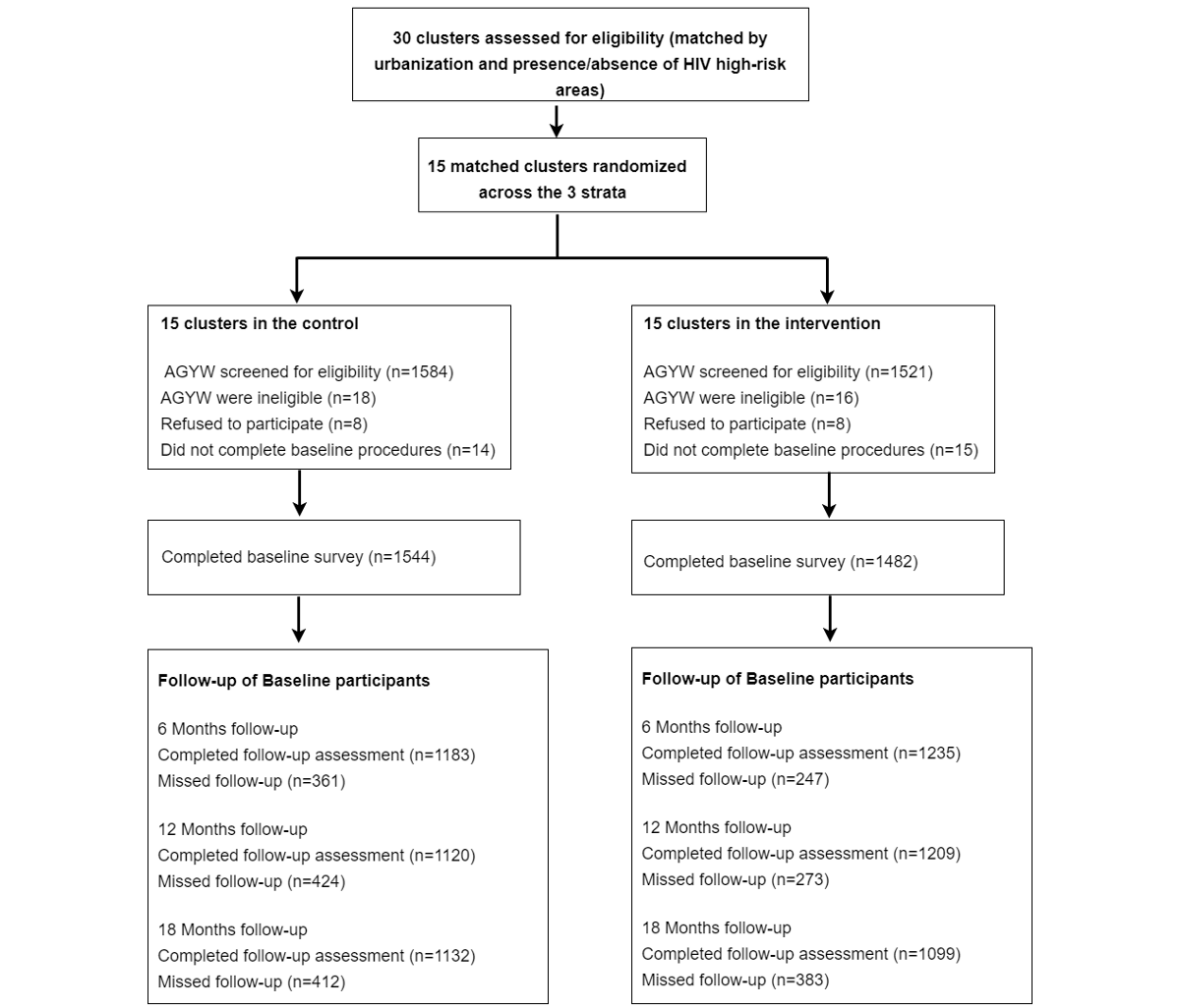
Trial profile for a cluster randomized trial among adolescent girls and young women in Shinyanga Region, Tanzania, in October 2017 to July 2019. AGYW: adolescent girls and young women; HSV-2: herpes simplex virus type 2.

Study recruitment took place between October 30, 2017 and December 1, 2017. Of the 3105 participants screened for eligibility (Figure 1), 3071 were eligible and 3055 consented/assented to study participation. Of these, 3026 (1482 in the intervention and 1544 in the control) participants completed baseline survey procedures and 2720 (1373 in the intervention and 1347 in the control) attended at least one follow-up study visit. Of those with follow-up data, 865 (443 in the intervention and 422 in the control) were infected with HSV-2 at baseline and, therefore, excluded from the HSV-2 longitudinal data analysis. Therefore, 1855 (930 in the intervention and 925 in the control) were included in the longitudinal analysis of the primary outcome contributing to 2524.2 personal years of observation (PYO) (1293.9 PYO in the intervention and 1230.3 PYO in the control) and had a retention of 61.3% (1855/3026; 930/1482, 62.7% in the intervention and 925/1544, 59.9% in the control). At baseline, the median age of the participants was 20 (IQR 18-22) years, and the intervention and control groups were similar for key sexual behavior variables, HIV prevalence, and HSV-2 prevalence. However, there were imbalances in the sociodemographic variables such as age, marital status, debt, and going to bed hungry (Table 1). The imbalance between the arms was adjusted for in the impact analysis.

**Table 1:** Baseline sociodemographic characteristics of the study participants in a cluster randomized trial among adolescent girls and young women in the Shinyanga Region, Tanzania, in October 2017 to July 2019.

Characteristics		Total (N=3026)	Intervention group (n=1482)	Control group (n=1544)
Age (years), median (IQR)		20 (18-22)	20 (18-22)	19 (17-22)
**Marital status, n (%)**				
	Single	1323 (43.7)	561 (37.9)	762 (49.4)
	Married	1514 (50)	828 (55.9)	686 (44.4)
	Separated, divorced, or widowed	189 (6.3)	93 (6.3)	96 (6.2)
**Educational status, n (%)**				
	No formal/not completed primary school	856 (28.3)	402 (27.1)	454 (29.4)
	Completed primary school	1609 (53.2)	790 (53.3)	819 (53)
	Complete/incomplete secondary school	561 (18.5)	290 (19.6)	271 (17.6)
Had emotional/psychological support, n (%)		2387 (78.9)	1200 (81)	1187 (76.9)
Had debt at the time of the survey, n (%)		1119 (37)	616 (41.6)	503 (32.6)
Lacked food (went to bed hungry past 4 weeks), n (%)		702 (23.2)	309 (20.9)	393 (25.5)
HIV-positive status^a^, n (%)		109 (3.6)	45 (3.1)	64 (4.2)
Herpes simplex virus type 2–positive status^b^, n (%)		956 (32)	478 (32.8)	478 (31.2)
Reported sex work (past 6 months)^c^, n (%)		387 (17)	183 (16.7)	204 (17.1)
Reported transactional sex (6 months)^c^, n (%)		694 (30.4)	316 (28.9)	378 (31.8)
Reported compensated sex (6 months)^c^, n (%)		792 (34.7)	365 (33.4)	427 (35.9)
Reported intergenerational sex (6 months)^c,d^, n (%)		283 (12.6)	127 (11.9)	156 (13.3)
Reported concurrent partnerships (6 months)^c^, n (%)		300 (13.1)	138 (12.6)	162 (13.6)
Reported condom use (nonmarital partner, 6 months)^c^, n (%)		3 (0.1)	1 (0.1)	2 (0.2)
Reported sexual partner violence (6 months)^c^, n (%)		817 (35.8)	377 (34.5)	440 (37)

^a^26 participants (18 in intervention and 8 in control) had either missing data or indeterminate results.

^b^36 participants (23 in intervention and 13 in control) had missing data.

^c^Restricted to those who were sexually active (n=2283), defined by self-reported vaginal or anal sex history (1093 in intervention and 1190 in control).

^d^48 participants (23 in intervention and 25 in control) had missing data.

### Study Implementation

Of the 1373 AGYW in the intervention arm included in the longitudinal data analysis, 465 (33.9%), 373 (27.2%), and 414 (30.2%) received cash transfer 3 times or less, 4 times, and over 4 times, respectively, while 121 (8.8%) did not disclose the number of times they received cash transfer. HSV-2 incidence was 5.7/100 PYO (ie, 5.7 girls become HSV-2 positive for 100 years of observation/57 seroconverts for every 1000 years), 9.1/100 PYO, 9.6/100 PYO, and 4.8/100 PYO among AGYW who received cash transfer 3 times or less, 4 times, over 4 times, and among those with missing information on the number of times they received cash transfer, respectively.

### Intervention Impact on HSV-2 Incidence and Other Outcomes

Two hundred incident HSV-2 infections were diagnosed in the study (98 in the control and 102 in the intervention), resulting in an annual incidence of 7.9/100 (95% CI 6.9/100-9.1/100) PYO. There was no significant difference in HSV-2 incidence between the study arms (adjusted hazard ratio 0.96, 95% CI 0.67-1.38; *P*=.83; Table 2), although location significantly modified the effect of cash transfer on HSV-2 incidence (effect modification *P*<.001). In urban and rural areas at high risk of HIV infection, cash transfer was associated with a nonsignificant increased hazard ratio adjusted for baseline confounders (Multimedia Appendix 1 and Multimedia Appendix 2). However, in rural areas with a low risk of HIV infection, the adjusted hazard ratio for incident HSV-2 infection in the cash transfer group was 0.45 (95% CI 0.29-0.71; *P*=.001; Multimedia Appendix 3).

There was no significant difference by study arm on HIV prevalence at follow-up and behavioral risk factors except for transactional sex and condom use with nonmarital sexual partners. The cash transfer intervention reduced the proportion of AGYW reporting transactional sex (adjusted OR 0.84, 0.73-0.96; *P*=.01), increased their savings (adjusted OR 1.87, 1.69-2.08; *P*<.001), and increased their utilization of community-based biomedical services (adjusted OR 2.10, 1.95-2.26; *P*<.001). There was no significant difference between the study groups in the proportion reporting compensated and intergenerational sex, sex work, and more than one sexual partner in the last 12 months. However, urbanization modified the effect of cash transfer intervention on savings (effect modification *P*<.001), utilization of biomedical services (*P*<.001), and sex work (*P*=.02). In urban areas, cash transfer was associated with a significant increase in reporting of sex work among those receiving the intervention compared to that in the control group adjusted for baseline confounders, although this was not the case in rural areas at high and low risks for HIV infection.

The 5 major items that AGYW spent their quarterly cash transfer on were starting a business (651/1373, 47.4%), toiletries (221/1373, 16.1%), savings (211/1373, 15.4%), food (143/1373, 10.4%), supporting dependents (114/1373, 8.3%), and other uses (33/1373, 2.4%). Only 3 social harm events were reported during the study—3 in the intervention and 0 in the control clusters—and were associated with minor teasing that cash transfer and phones provided to facilitate cash transfer may be associated with Freemasonry.

**Table 2:** Effect of cash transfer on primary and secondary outcomes in a cluster randomized trial among adolescent girls and young women in Shinyanga Region, Tanzania, in October 2017 to July 2019.

			Overall				Urban stratum						Rural high-risk stratum						Rural low-risk stratum			
			Intervention group		Control group		Intervention group			Control group		Intervention group				Control group		Intervention group				Control group
**HSV-2 new cases out of total at risk, n/N (%)**			102/930 (11)		98/925 (10.6)		29/310 (9.4)			17/327 (5.2)		45/322 (14)				21/261 (8)		28/298 (9.4)				60/337 (17.8)
	Time of follow-up (years)	1293.9		1230.3		426.0			438.9		450.0				346.3		417.9				445.1	
	HSV-2 incidence (per 100 personal years of observation)	7.9		8.0		6.8			3.9		10.0				6.1		6.7				13.5	
	Hazard ratio^a^	0.96 (0.67-1.38)				1.55 (0.84-2.84)						1.60 (0.95-2.71)						0.45 (0.29-0.71)				
	Hazard ratio *P* value	.83				.16						.08						.001				
	*P* value for interaction	<.001				—^b^			—		—				—		—				—	
**New HIV cases out of total at risk, n/N (%)**			11/1429 (0.8)		9/1478 (0.6)		—			—		—				—		—				—
	Time of follow-up (years)	1954.7		1811.8		—			—		—				—		—				—	
	HIV incidence (per 1000 personal years of observation)	5.6		5.0		—			—		—				—		—				—	
	Hazard ratio^a^	0.78 (0.40-1.53)				—			—		—				—		—				—	
	Hazard ratio *P* value	.47				—			—		—				—		—				—	
**HIV prevalence (follow-up), n/N (%)**			58/1466 (4)		74/1537 (4.8)		—			—		—				—		—				—
	Risk ratio^a^	0.75 (0.52-1.08)				—			—		—				—		—				—	
	Risk ratio *P* value	.13				—			—		—				—		—				—	
**Compensated sex, n/N (%)**			552/1373 (40.2)		580/1347 (43.1)		N/A^c^			N/A		N/A				N/A		N/A				N/A
	Odds ratio^a^	0.91 (0.80-1.04)				N/A			N/A		N/A				N/A		N/A				N/A	
	Odds ratio *P* value	.15				N/A			N/A		N/A				N/A		N/A				N/A	
	*P* value for interaction	.46				—			—		—				—		—				—	
**Intergenerational sex, n/N (%)**			374/1373 (27.2)		362/1347 (26.9)		N/A			N/A		N/A				N/A		N/A				N/A
	Odds ratio^a^	0.91 (0.77-1.07)				N/A			N/A		N/A				N/A		N/A				N/A	
	Odds ratio *P* value	.25				N/A			N/A		N/A				N/A		N/A				N/A	
	*P* value for interaction	.88				—			—		—				—		—				—	
**Transactional sex, n/N (%)**			486/1373 (35.4)		533/1347 (39.6)		N/A			N/A		N/A				N/A		N/A				N/A
	Odds ratio^a^	0.84 (0.73-0.96)				N/A			N/A		N/A				N/A		N/A				N/A	
	Odds ratio *P* value	.01				N/A			N/A		N/A				N/A		N/A				N/A	
	*P* value for interaction	.50				—			—		—				—		—				—	
**Condom use (nonmarital)^d^, n/N (%)**			867/1284 (67.5)		738/1205 (61.2)		N/A			N/A		N/A				N/A		N/A				N/A
	Odds ratio^a^	1.28 (1.16-1.42)				N/A			N/A		N/A				N/A		N/A				N/A	
	Odds ratio *P* value	<.001				N/A			N/A		N/A				N/A		N/A				N/A	
	*P* value for interaction	.22				—			—		—				—		—				—	
**>1 sexual partner (12 months), n/N** **(%)**			211/1373 (15.4)		207/1347 (15.4)		N/A			N/A		N/A				N/A		N/A				N/A
	Odds ratio^a^	0.97 (0.79-1.20)				N/A			N/A		N/A				N/A		N/A				N/A	
	Odds ratio *P* value	.80				N/A			N/A		N/A				N/A		N/A				N/A	
	*P* value for interaction	.12				—			—		—				—		—				—	
**Savings, n/N (%)**			1275/1373 (92.9)		1140/1347 (84.6)		402/458 (87.8)			379/437 (86.7)		462/480 (96.3)				338/421 (80.3)		411/435 (94.5)				423/489 (86.5)
	Odds ratio^a^	1.87 (1.69-2.08)				0.92 (0.76-1.11)						3.13 (2.63-3.72)						2.28 (1.91-2.71)				
	Odds ratio *P* value	<.001				.40						<.001						<.001				
	*P* value for interaction	<.001				—			—		—				—		—				—	
**Used biomedical services, n/N (%)**			1161/1373 (84.6)		820/1347 (60.9)		393/458 (85.8)			225/437 (51.5)		362/480 (75.4)				275/421 (65.3)		406/435 (93.3)				320/489 (65.4)
	Odds ratio^a^	2.10 (1.95-2.26)				2.63 (2.28-3.03)						1.35 (1.18-1.54)						2.42 (2.15-2.72)				
	Odds ratio *P* value	<.001				<.001						<.001						<.001				
	*P* value for interaction	<.001				—			—		—				—		—				—	
**Sexual partner violence, n/N (%)**			711/1373 (51.8)		685/1347 (50.9)		N/A			N/A		N/A				N/A		N/A				N/A
	Odds ratio^a^	0.94 (0.83-1.06)				N/A			N/A		N/A				N/A		N/A				N/A	
	Odds ratio *P* value	.29				N/A			N/A		N/A				N/A		N/A				N/A	
	*P* value for interaction	.83				—			—		—				—		—				—	
**Sex work, n/N (%)**			310/1373 (22.6)		315/1347 (23.4)		87/458 (19)			58/437 (13.3)		114/480 (23.8)				121/421 (28.7)		109/435 (25.1)				136/489 (27.8)
	Odds ratio^a^	1.06 (0.89-1.26)				1.66 (1.16-2.36)						0.90 (0.68-1.19)						0.92 (0.69-1.22)				
	Odds ratio *P* value	.53				.005						.47						.56				
	*P* value for interaction	.02				—		—				—		—				—		—		

^a^Adjusted for matching pairs and variables that were significantly different between the arms at baseline.

^b^Not available.

^c^N/A: not applicable.

^d^Restricted to adolescent girls and young women who had follow-up data and reported sexual activity in the last 6 months.

## Discussion

Overall, this study observed no significant effect of the quarterly cash transfer on HSV-2 incidence among out-of-school AGYW after 18 months of intervention. However, cash transfer was associated with a reduced incidence of HSV-2 infection in rural communities at low risk of HIV infections but not in urban and rural communities with high risk of HIV infections. This difference may be because of factors in urban and rural high-risk communities that may affect the effectiveness of cash transfer as a structural intervention for HIV infection. These factors may include less poverty, high mobility, and migration in the urban areas and rural high-risk communities where there are mines compared to rural low-risk areas. For instance, almost 81% of the low-income population in Tanzania reside in rural areas, depending on subsistence agriculture for their livelihood [45]. In 2015, 20.8% of the rural households were clustered in the lowest-income quartile against 3.7% of the urban households [46]. In this study, only 12% (11% urban, 9% rural high-risk villages vs 15% rural low-risk villages; *P*<.001) of the AGYW were living in households supported by the Government of Tanzania social action fund, which targets the lowest-income and vulnerable households [47]. Previous studies have shown that cash transfer has a significant impact on low-income communities as compared to that on high-income communities [48,49]. Since AGYW in rural areas have less access to jobs and other income-generating activities, they may engage in transactional sex to fulfil basic needs [50,51]. It is therefore likely that cash transfer was associated with a reduced incidence of HSV-2 infection in rural low-risk communities because a high proportion of AGYW in these communities were able to meet their basic needs through cash transfer and thus reduce risky sexual behavior [29]. Moreover, 10.6% (107/1014) of AGYW in urban areas, 10.6% (108/1021) of AGYW in rural high-risk areas, and 6.6% (65/991) of AGYW in rural low-risk areas who participated in the baseline survey had migrated to other distant areas and were not seen in the subsequent follow-up rounds even after extensive tracing. Evidence suggests that mobile populations and migrants tend to be more vulnerable to sexually transmitted infections than nonmigrating populations [52,53]. Mining areas and towns surrounding mines attract a large number of male miners, thereby affecting the age-specific sex ratio in the area [54], altering the number of available sexual partners [55] and sexual networks [54,56] and placing AGYW in an environment conducive to practice transactional sex [56]. In this study, the cash transfer intervention was associated with higher reporting of sex work in urban areas and higher HSV-2 incidence rates among AGYW with more exposure due to higher cash transfer.

Urban and rural communities with many small-scale mining activities that put them at high risk of HIV infection have better asset ownership than rural communities [46,57]. In control communities, the incidence of HSV-2 was 13.5/100 PYO in the rural low-risk stratum, 6.1/100 PYO in the rural high-risk stratum, and 3.9/100 PYO in the urban areas. The high incidence in rural remote villages may be because of small densely connected sexual networks, which have been shown to be highly effective in spreading viral sexually transmitted infections [58,59]. Owing to the high prevalence of HSV-2 at baseline in these communities, AGYW selecting new sexual partners in rural areas are more likely to choose a partner who is HSV-2 positive because of their remoteness and small population size [58,59], unlike in rural high-risk and urban areas where there are large-scale migrations and mobility. Finally, AGYW in remote rural villages may also have limited access to health care, especially the management of viral sexually transmitted infections, which may elevate their risk of HSV-2 infection [60].

The cash transfer intervention did not reduce HIV incidence in the intervention arm compared to that in the control arm. Most of the evidences related to the impact of cash transfer on HIV prevention among AGYW in sub-Saharan Africa have been reported in studies conducted among adolescent schoolgirls and mostly in rural areas [22]. Keeping young girls in school is associated with a reduced risk of HIV infection [29]. Our study was conducted among out-of-school AGYW who were economically vulnerable and therefore more likely to be at high risk of HIV infections compared to economically nonvulnerable AGYW. The economic vulnerability of AGYW who dropped out of school may be caused by factors that led them to leave school, such as lack of financial support, loss of parents, sick parents, pregnancy, or early marriage [61]. Thus, the cash amount provided through the cash transfer intervention may not have been sufficient to bring the AGYW out of the risk behavior.

The baseline findings from this study showed HSV-2 prevalence of 32% (956/2990), which is comparable to that reported in earlier studies conducted in similar settings [43,62,63]. HSV-2 infection increases the risk of acquiring HIV infection up to 5-fold [64]. The high baseline HSV-2 prevalence is probably because of several large-scale and small-scale gold mining activities with a large number of men attracting many economically vulnerable young women, creating a niche for transactional sex and sexual mixing where the partnership is formed between partners with different HIV risk profiles [65]. The high HSV-2 prevalence observed in this study indicates the need for continued targeted prevention efforts among AGYW to saturate the region with interventions to reduce new infections. Our study contributes to the literature on cash transfer, as it reports the synergetic effect of cash transfer as a social protection scheme along with CHP among out-of-school girls where HIV infection is the highest. To our knowledge, only the adolescent girls’ initiative study in Kenya has been implemented in this group in sub-Saharan Africa [66]. Previous cash transfer interventions have produced mixed results, with some demonstrating impact on preventing sexually transmitted infections among school girls by delaying sexual activity [22,24,25]. Our study shows that cash transfer programs targeting the low-income and highly vulnerable populations in rural areas are more likely to reduce risky sexual behavior among AGYW. However, more studies are needed to evaluate the effect of cash transfer among out-of-school AGYW aged 15-24 years in different settings and the different amounts of cash transfer because of high HIV infection rates in this subpopulation.

This study has several strengths. First, we used ACASI to collect sexual behavior data and other sensitive data. Studies comparing face-to-face interviews with ACASI have reported that respondents are more likely to be open and honest when using ACASI in reporting sensitive information [67,68]. Second, to assess the effect of cash transfer, our study collected longitudinal data on biomedical, behavioral, and structural interventions on a large sample of AGYW in urban and rural areas who were out of school and therefore vulnerable to HIV infections. Third, the CHP intervention package was developed after broad consultation and engagement with local leaders; the Ministry of Health, Community Development, Gender, Elderly and Children; Government of Tanzania social action fund; National AIDS Control Program; Tanzania Commission for HIV/AIDS; mobile communication companies; and CSOs serving AGYW and AGYW representatives among other stakeholders. These dialogues led to developing a tailored and prioritized CHP intervention package to AGYW offered by the Sauti project, including the cash transfer amount and payment modality. The involvement of CSOs serving AGYW and AGYW representatives was crucial in advising on the content of the study materials and data collection techniques such as ACASI. CSOs were crucial in the recruitment of AGYW for CHP interventions alongside the recruitment of study participants and monitoring of study activities and tracing of study participants. CSOs were also involved in the dissemination of the study findings at the community and regional levels. A limitation of this study is that the clusters were selected only from the Shinyanga Region, as the prevalence of HIV in this region was higher than the national average, and thus, these findings may not be generalizable to other study regions with lower HIV prevalence in Tanzania.

In conclusion, this trial showed no significant impact of the cash transfer intervention on HSV-2 incidence among AGYW in Tanzania. Although the intervention appears to have reduced HSV-2 incidence among AGYW in rural low-risk communities, this effect was not observed in urban high-risk communities. Factors such as less poverty and more asset ownership in urban and rural high-risk (mining) communities may have undermined the effect of cash transfer.

## Appendix

Multimedia Appendix 1 Kaplan-Meier curve for herpes simplex virus type 2 seroconversion in urban areas in a cluster randomized trial among adolescent girls and young women in Shinyanga region, Tanzania, in October 2017-July 2019.

Multimedia Appendix 2 Kaplan-Meier curve for herpes simplex virus type 2 seroconversion in rural areas at high HIV risk in a cluster randomized trial among adolescent girls and young women in Shinyanga region, Tanzania, in October 2017-July 2019.

Multimedia Appendix 3 Kaplan-Meier curve for herpes simplex virus type 2 seroconversion in rural areas at low HIV risk in a cluster randomized trial among adolescent girls and young women in Shinyanga region, Tanzania, in October 2017-July 2019.

Multimedia Appendix 4 CONSORT EHEALTH checklist (V 1.6.1).

## Abbreviations

ACASI: audio computer-assisted self-interview
AGYW: adolescent girls and young women
CHP: combination HIV prevention
CONSORT: Consolidated Standards of Reporting Trials
CSO: civil society organization
DREAMS: Determined, Resilient, Empowered, AIDS-free, Mentored, and Safe
HSV-2: herpes simplex virus type 2
OR: odds ratio
PYO: personal years of observation
SBCC: social and behavior change communication
WORTH+: Women Organizing Resources Together plus
