# Supplementary figures and images for: Effectiveness of Cash Transfer Delivered Along With Combination HIV Prevention Interventions in Reducing the Risky Sexual Behavior of Adolescent Girls and Young Women in Tanzania: Cluster Randomized Controlled Trial
Source: JMIR Public Health Surveill. 2022 Sep 19;8(9):e30372. doi: 10.2196/30372 (PMC9531008; doi:10.2196/30372)

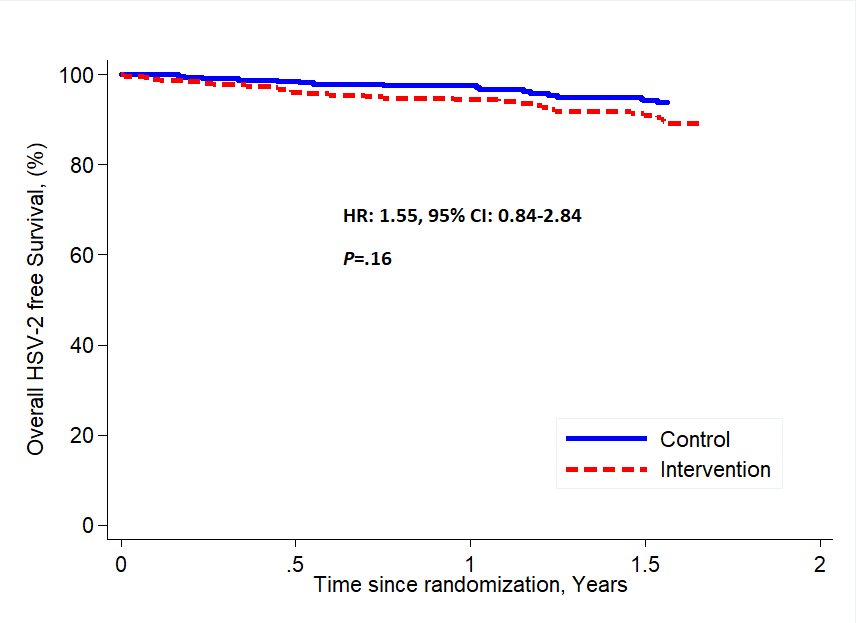

Supplement: Multimedia Appendix 1 [file publichealth_v8i9e30372_app1.png]

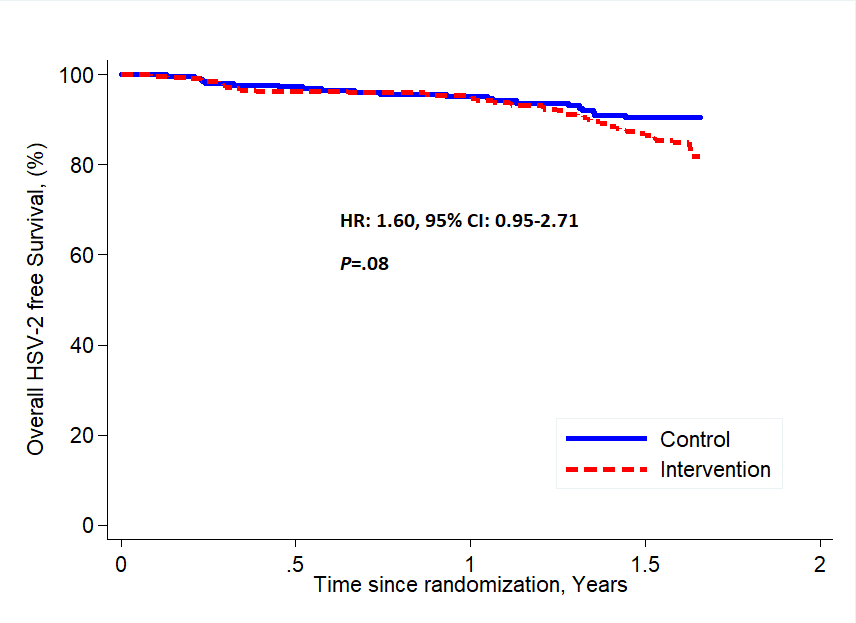

Supplement: Multimedia Appendix 2 [file publichealth_v8i9e30372_app2.png]

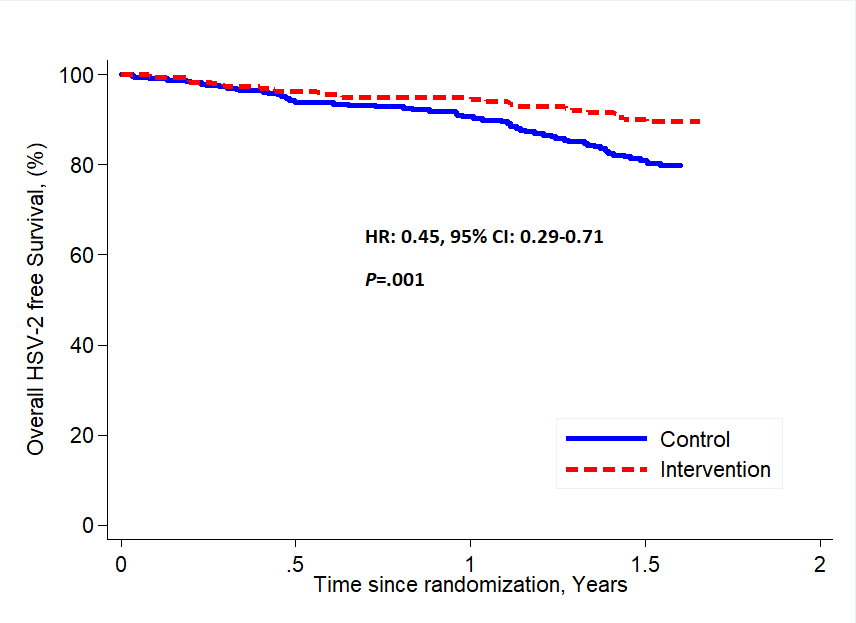

Supplement: Multimedia Appendix 3 [file publichealth_v8i9e30372_app3.png]
