# Supplementary material for: Effectiveness of Cash Transfer Delivered Along With Combination HIV Prevention Interventions in Reducing the Risky Sexual Behavior of Adolescent Girls and Young Women in Tanzania: Cluster Randomized Controlled Trial
Source: JMIR Public Health Surveill. 2022 Sep 19;8(9):e30372. doi: 10.2196/30372 (PMC9531008; doi:10.2196/30372)
Supplement: Multimedia Appendix 4 [file publichealth_v8i9e30372_app4.pdf]

CONSORT-EHEALTH (V 1.6.1) - Submission/Publication Form

The CONSORT-EHEALTH checklist is intended for authors of randomized trials evaluating web-based and Internet-based applications/interventions, including mobile interventions, electronic games (incl multiplayer games), social media, certain telehealth applications, and other interactive and/or networked electronic applications. Some of the items (e.g. all subitems under item 5 - description of the intervention) may also be applicable for other study designs.

The goal of the CONSORT EHEALTH checklist and guideline is to be
a) a guide for reporting for authors of RCTs,
b) to form a basis for appraisal of an ehealth trial (in terms of validity)

CONSORT-EHEALTH items/subitems are MANDATORY reporting items for studies published in the Journal of Medical Internet Research and other journals / scientific societies endorsing the checklist.

Items numbered 1., 2., 3., 4a., 4b etc are original CONSORT or CONSORT-NPT (non-pharmacologic treatment) items.
Items with Roman numerals (i., ii, iii, iv etc.) are CONSORT-EHEALTH extensions/clarifications.

As the CONSORT-EHEALTH checklist is still considered in a formative stage, we would ask that you also RATE ON A SCALE OF 1-5 how important/useful you feel each item is FOR THE PURPOSE OF THE CHECKLIST and reporting guideline (optional).

Mandatory reporting items are marked with a red *.
In the textboxes, either copy & paste the relevant sections from your manuscript into this form - please include any quotes from your manuscript in QUOTATION MARKS,
or answer directly by providing additional information not in the manuscript, or elaborating on why the item was not relevant for this study.

YOUR ANSWERS WILL BE PUBLISHED AS A SUPPLEMENTARY FILE TO YOUR PUBLICATION IN JMIR AND ARE CONSIDERED PART OF YOUR PUBLICATION (IF ACCEPTED).
Please fill in these questions diligently. Information will not be copyedited, so please use proper spelling and grammar, use correct capitalization, and avoid abbreviations.

DO NOT FORGET TO SAVE AS PDF _AND_ CLICK THE SUBMIT BUTTON SO YOUR ANSWERS ARE IN OUR DATABASE !!!

Citation Suggestion (if you append the pdf as Appendix we suggest to cite this paper in the caption):
Eysenbach G, CONSORT-EHEALTH Group

CONSORT-EHEALTH: Improving and Standardizing Evaluation Reports of Web-based and Mobile Health Interventions
J Med Internet Res 2011;13(4):e126
URL: http://www.jmir.org/2011/4/e126/
doi: 10.2196/jmir.1923
PMID: 22209829

wmwita51@gmail.com (not shared) Switch accounts

Draft saved

*Required

Your name *

First Last

Mwita Wambura

Primary Affiliation (short), City, Country *

University of Toronto, Toronto, Canada

NIMR, Mwanza, Tanzania

Your e-mail address *

abc@gmail.com

wmwita51@gmail.com

Title of your manuscript *

Provide the (draft) title of your manuscript.

Effectiveness of Cash Transfer Delivered Along With Combination HIV Prevention Interventions in Reducing the Risky Sexual Behavior of Adolescent Girls and Young Women in Tanzania: Cluster Randomized Controlled Trial

Name of your App/Software/Intervention *

If there is a short and a long/alternate name, write the short name first and add the long name in brackets.

Cash transfer

Evaluated Version (if any)

e.g. "V1", "Release 2017-03-01", "Version 2.0.27913"

Your answer

Language(s) *

What language is the intervention/app in? If multiple languages are available, separate by comma (e.g. "English, French")

Swahili

URL of your Intervention Website or App

e.g. a direct link to the mobile app on app in appstore (itunes, Google Play), or URL of the website. If the intervention is a DVD or hardware, you can also link to an Amazon page.

Your answer

URL of an image/screenshot (optional)

Your answer

Accessibility *

Can an enduser access the intervention presently?

- ○ access is free and open
- ○ access only for special usergroups, not open
- ○ access is open to everyone, but requires payment/subscription/in-app purchases
- ○ app/intervention no longer accessible
- ◉ Other: The intervention is cash transfer given in presence of other combinati

Primary Medical Indication/Disease/Condition *

e.g. "Stress", "Diabetes", or define the target group in brackets after the condition, e.g. "Autism (Parents of children with)", "Alzheimers (Informal Caregivers of)"

HIV infection

Primary Outcomes measured in trial *

comma-separated list of primary outcomes reported in the trial

HSV-2 incidence

Secondary/other outcomes

Are there any other outcomes the intervention is expected to affect?

HIV prevalence at follow-up, self-reported intergenerational sex and self-reported compensated sex

Recommended "Dose" *

What do the instructions for users say on how often the app should be used?

- ○ Approximately Daily
- ○ Approximately Weekly
- ○ Approximately Monthly
- ○ Approximately Yearly
- ○ "as needed"
- ◉ Other: Unconditional cash transfer in quarterly instalments of 70,000 Tanzan

Approx. Percentage of Users (starters) still using the app as recommended after 3 months *

- (selected) unknown / not evaluated
- 0-10%
- 11-20%
- 21-30%
- 31-40%
- 41-50%
- 51-60%
- 61-70%
- 71%-80%
- 81-90%
- 91-100%
- Other:

Overall, was the app/intervention effective? *

- yes: all primary outcomes were significantly better in intervention group vs control
- partly: SOME primary outcomes were significantly better in intervention group vs control
- (selected) no statistically significant difference between control and intervention
- potentially harmful: control was significantly better than intervention in one or more outcomes
- inconclusive: more research is needed
- Other:

Article Preparation Status/Stage *

At which stage in your article preparation are you currently (at the time you fill in this form)

- ( ) not submitted yet - in early draft status
- ( ) not submitted yet - in late draft status, just before submission
- ( ) submitted to a journal but not reviewed yet
- ( ) submitted to a journal and after receiving initial reviewer comments
- (x) submitted to a journal and accepted, but not published yet
- ( ) published
- ( ) Other:

Journal *

If you already know where you will submit this paper (or if it is already submitted), please provide the journal name (if it is not JMIR, provide the journal name under "other")

- ( ) not submitted yet / unclear where I will submit this
- ( ) Journal of Medical Internet Research (JMIR)
- ( ) JMIR mHealth and UHealth
- ( ) JMIR Serious Games
- ( ) JMIR Mental Health
- (x) JMIR Public Health
- ( ) JMIR Formative Research
- ( ) Other JMIR sister journal
- ( ) Other:

Is this a full powered effectiveness trial or a pilot/feasibility trial? *

- ○ Pilot/feasibility
- ◉ Fully powered

Manuscript tracking number *

If this is a JMIR submission, please provide the manuscript tracking number under "other" (The ms tracking number can be found in the submission acknowledgement email, or when you login as author in JMIR. If the paper is already published in JMIR, then the ms tracking number is the four-digit number at the end of the DOI, to be found at the bottom of each published article in JMIR)

- ○ no ms number (yet) / not (yet) submitted to / published in JMIR
- ◉ Other: ms#30372

TITLE AND ABSTRACT

1a) TITLE: Identification as a randomized trial in the title

1a) Does your paper address CONSORT item 1a? *

I.e does the title contain the phrase "Randomized Controlled Trial"? (if not, explain the reason under "other")

- ◉ yes
- ○ Other:

1a-i) Identify the mode of delivery in the title

Identify the mode of delivery. Preferably use “web-based” and/or “mobile” and/or “electronic game” in the title. Avoid ambiguous terms like “online”, “virtual”, “interactive”. Use “Internet-based” only if Intervention includes non-web-based Internet components (e.g. email), use “computer-based” or “electronic” only if offline products are used. Use “virtual” only in the context of “virtual reality” (3-D worlds). Use “online” only in the context of “online support groups”. Complement or substitute product names with broader terms for the class of products (such as “mobile” or “smart phone” instead of “iphone”), especially if the application runs on different platforms.

	1	2	3	4	5	
subitem not at all important	(●)	( )	( )	( )	( )	essential

Clear selection

Does your paper address subitem 1a-i? *

Copy and paste relevant sections from manuscript title (include quotes in quotation marks "like this" to indicate direct quotes from your manuscript), or elaborate on this item by providing additional information not in the ms, or briefly explain why the item is not applicable/relevant for your study

This is not applicable to the current study

1a-ii) Non-web-based components or important co-interventions in title

Mention non-web-based components or important co-interventions in title, if any (e.g., “with telephone support”).

	1	2	3	4	5	
subitem not at all important	(●)	( )	( )	( )	( )	essential

Clear selection

Does your paper address subitem 1a-ii?

Copy and paste relevant sections from manuscript title (include quotes in quotation marks "like this" to indicate direct quotes from your manuscript), or elaborate on this item by providing additional information not in the ms, or briefly explain why the item is not applicable/relevant for your study

Your answer

1a-iii) Primary condition or target group in the title

Mention primary condition or target group in the title, if any (e.g., “for children with Type I Diabetes”) Example: A Web-based and Mobile Intervention with Telephone Support for Children with Type I Diabetes: Randomized Controlled Trial

	1	2	3	4	5	
subitem not at all important	○	○	○	○	●	essential

Clear selection

Does your paper address subitem 1a-iii? *

Copy and paste relevant sections from manuscript title (include quotes in quotation marks "like this" to indicate direct quotes from your manuscript), or elaborate on this item by providing additional information not in the ms, or briefly explain why the item is not applicable/relevant for your study

"Effectiveness of Cash Transfer Delivered Along With Combination HIV Prevention Interventions in Reducing the Risky Sexual Behavior of Adolescent Girls and Young Women in Tanzania: Cluster Randomized Controlled Trial"

1b) ABSTRACT: Structured summary of trial design, methods, results, and conclusions

NPT extension: Description of experimental treatment, comparator, care providers, centers, and blinding status.

1b-i) Key features/functionalities/components of the intervention and comparator in the METHODS section of the ABSTRACT

Mention key features/functionalities/components of the intervention and comparator in the abstract. If possible, also mention theories and principles used for designing the site. Keep in mind the needs of systematic reviewers and indexers by including important synonyms. (Note: Only report in the abstract what the main paper is reporting. If this information is missing from the main body of text, consider adding it)

	1	2	3	4	5	
subitem not at all important	○	○	○	○	◉	essential

Clear selection

Does your paper address subitem 1b-i? *

Copy and paste relevant sections from the manuscript abstract (include quotes in quotation marks "like this" to indicate direct quotes from your manuscript), or elaborate on this item by providing additional information not in the ms, or briefly explain why the item is not applicable/relevant for your study

"Poverty and social inequality exacerbate HIV risk among adolescent girls and young women (AGYW) in sub-Saharan Africa. Cash transfers can influence the structural determinants of health, thereby reducing HIV risk"
"This study assessed the effectiveness of cash transfer delivered along with combination HIV prevention (CHP) interventions in reducing the risky sexual behavior of AGYW in Tanzania. The incidence of herpes simplex virus type 2 (HSV-2) infection was used as a proxy for sexual risk behavior".

1b-ii) Level of human involvement in the METHODS section of the ABSTRACT

Clarify the level of human involvement in the abstract, e.g., use phrases like “fully automated” vs. “therapist/nurse/care provider/physician-assisted” (mention number and expertise of providers involved, if any). (Note: Only report in the abstract what the main paper is reporting. If this information is missing from the main body of text, consider adding it)

	1	2	3	4	5	
subitem not at all important	○	○	○	○	◉	essential

Clear selection

Does your paper address subitem 1b-ii?

Copy and paste relevant sections from the manuscript abstract (include quotes in quotation marks "like this" to indicate direct quotes from your manuscript), or elaborate on this item by providing additional information not in the ms, or briefly explain why the item is not applicable/relevant for your study

"Participants completed an Audio Computer-Assisted Self-Interview, HIV counselling and testing, and HSV-2 testing at baseline and during follow-up visits at 6, 12 and 18 months."

1b-iii) Open vs. closed, web-based (self-assessment) vs. face-to-face assessments in the METHODS section of the ABSTRACT

Mention how participants were recruited (online vs. offline), e.g., from an open access website or from a clinic or a closed online user group (closed usergroup trial), and clarify if this was a purely web-based trial, or there were face-to-face components (as part of the intervention or for assessment). Clearly say if outcomes were self-assessed through questionnaires (as common in web-based trials). Note: In traditional offline trials, an open trial (open-label trial) is a type of clinical trial in which both the researchers and participants know which treatment is being administered. To avoid confusion, use “blinded” or “unblinded” to indicated the level of blinding instead of “open”, as “open” in web-based trials usually refers to “open access” (i.e. participants can self-enrol). (Note: Only report in the abstract what the main paper is reporting. If this information is missing from the main body of text, consider adding it)

	1	2	3	4	5	
subitem not at all important	○	○	○	○	◉	essential

Clear selection

Does your paper address subitem 1b-iii?

Copy and paste relevant sections from the manuscript abstract (include quotes in quotation marks "like this" to indicate direct quotes from your manuscript), or elaborate on this item by providing additional information not in the ms, or briefly explain why the item is not applicable/relevant for your study

"A cluster randomized controlled trial was conducted in 15 matched pairs of communities (1:1 intervention to control) across 3 strata (urban, rural high-risk, and rural low-risk populations) of the Shinyanga Region, Tanzania. The target population was out-of-school AGYW aged 15-23 years who had completed 10-hour sessions of social and behavior change communication. Eligible communities were randomly assigned to receive CHP along with cash transfer quarterly (intervention group) or solely CHP interventions (control group) with no masking. Study recruitment and baseline survey were conducted between October 30, 2017 and December 1, 2017. Participants completed an audio computer-assisted self-interview, HIV counselling and testing, and HSV-2 testing at baseline and during follow-up visits at 6, 12, and 18 months after the baseline survey. A Cox proportional hazards model with random effects specified at the level of clusters (shared frailty) adjusted for matching pairs and other baseline imbalances was fitted to assess the effects of cash transfer on the incidence of HSV-2 infection (primary outcome). Secondary outcomes included HIV prevalence at follow-up, self-reported intergenerational sex, and self-reported compensated sex. All secondary outcomes were measured at each study visit".

1b-iv) RESULTS section in abstract must contain use data

Report number of participants enrolled/assessed in each group, the use/uptake of the intervention (e.g., attrition/adherence metrics, use over time, number of logins etc.), in addition to primary/secondary outcomes. (Note: Only report in the abstract what the main paper is reporting. If this information is missing from the main body of text, consider adding it)

	1	2	3	4	5	
subitem not at all important	○	○	○	○	◉	essential

Clear selection

Does your paper address subitem 1b-iv?

Copy and paste relevant sections from the manuscript abstract (include quotes in quotation marks "like this" to indicate direct quotes from your manuscript), or elaborate on this item by providing additional information not in the ms, or briefly explain why the item is not applicable/relevant for your study

"Of the 3026 AGYW enrolled in the trial (1482 in the intervention and 1544 in the control), 2720 AGYW (1373 in the intervention and 1347 in the control) were included in the final analysis. Overall, HSV-2 incidence was not significantly different at all follow-up points between the study arms in the adjusted analysis (hazard ratio 0.96, 95% CI 0.67-1.38; P=.83). However, HSV-2 incidence was significantly lower in the rural low-risk populations who received the cash transfer intervention (hazard ratio 0.45, 95% CI 0.29-0.71; P=.001), adjusted for potential confounders".

1b-v) CONCLUSIONS/DISCUSSION in abstract for negative trials

Conclusions/Discussions in abstract for negative trials: Discuss the primary outcome - if the trial is negative (primary outcome not changed), and the intervention was not used, discuss whether negative results are attributable to lack of uptake and discuss reasons. (Note: Only report in the abstract what the main paper is reporting. If this information is missing from the main body of text, consider adding it)

	1	2	3	4	5	
subitem not at all important	○	○	○	○	◉	essential

Clear selection

Does your paper address subitem 1b-v?

Copy and paste relevant sections from the manuscript abstract (include quotes in quotation marks "like this" to indicate direct quotes from your manuscript), or elaborate on this item by providing additional information not in the ms, or briefly explain why the item is not applicable/relevant for your study

"Although this trial showed no significant impact of the cash transfer intervention on HSV-2 incidence among AGYW overall, the intervention significantly reduced HSV-2 incidence among AGYW in rural low-risk communities. Factors such as lesser poverty and more asset ownership in urban and rural high-risk communities may have undermined the impact of cash transfer".

INTRODUCTION

2a) In INTRODUCTION: Scientific background and explanation of rationale

2a-i) Problem and the type of system/solution

Describe the problem and the type of system/solution that is object of the study: intended as stand-alone intervention vs. incorporated in broader health care program? Intended for a particular patient population? Goals of the intervention, e.g., being more cost-effective to other interventions, replace or complement other solutions? (Note: Details about the intervention are provided in “Methods” under 5)

	1	2	3	4	5	
subitem not at all important	○	○	○	○	◉	essential

Clear selection

Does your paper address subitem 2a-i? *

Copy and paste relevant sections from the manuscript (include quotes in quotation marks "like this" to indicate direct quotes from your manuscript), or elaborate on this item by providing additional information not in the ms, or briefly explain why the item is not applicable/relevant for your study

"Adolescent girls and young women (AGYW) aged 15-24 years in eastern and southern regions of Africa have high rates of HIV infection [1-4]. In Tanzania, the HIV incidence was 0.14% (95% CI 0%-0.31%) and 0% (95% CI 0%-0.23%) in 2016 among AGYW and adolescent boys and young men, respectively [5]. Targeted HIV prevention efforts among AGYW are crucial to realize an HIV-free generation [6]".

2a-ii) Scientific background, rationale: What is known about the (type of) system

Scientific background, rationale: What is known about the (type of) system that is the object of the study (be sure to discuss the use of similar systems for other conditions/diagnoses, if appropiate), motivation for the study, i.e. what are the reasons for and what is the context for this specific study, from which stakeholder viewpoint is the study performed, potential impact of findings [2]. Briefly justify the choice of the comparator.

	1	2	3	4	5	
subitem not at all important	○	○	○	○	◉	essential

Clear selection

Does your paper address subitem 2a-ii? *

Copy and paste relevant sections from the manuscript (include quotes in quotation marks "like this" to indicate direct quotes from your manuscript), or elaborate on this item by providing additional information not in the ms, or briefly explain why the item is not applicable/relevant for your study

"Interactions among biological [7-9], behavioral [10-15], and structural determinants of health [10,16] have been associated with higher HIV infection risks in AGYW, with poverty [10,16] and gender inequality [10,17] exacerbating their vulnerability. AGYW from low-income households are likely to engage in transactional sex [12,15] to cater to their daily subsistence and are less likely to negotiate safer sexual practices [9,10,18]. This highlights the need for incorporating structural interventions in combination with biomedical and behavioral approaches for impactful HIV prevention among AGYW [19-21]. Cash transfer as a structural intervention has shown promising results, with some studies demonstrating its impact on the prevention of sexually transmitted infections and delaying marriage and childbearing [22-25]. Of the 8 cash transfer studies conducted in sub-Saharan Africa with sexually transmitted infections as an outcome, 4 reported a significant reduction; cash transfer provided to in-school AGYW aged 13-22 years and their parents reduced HIV prevalence by 64% (95% CI 9%-86%) and herpes simplex virus type 2 (HSV-2) prevalence by 76% (95% CI 35%-91%) [22]. Short-term financial incentives to engage in safe sex in Lesotho reduced HIV incidence among males and females aged 18-30 years by 25% (95% CI 3%-42%) [25]. Similarly, another financial incentive to engage in safe sex in Eswatini, which was conditional on staying sexually transmitted infection–negative, reduced HIV incidence among schoolgirls by 23% (95% CI 1%-40%) compared to those not eligible for educational cash transfer [23,26]. A South African study [27,28] showed reduced HSV-2 incidence by 30% (95% CI 14%-43%) among school-based girls and boys but had too few new infections for HIV incidence analysis. Other studies [29,30] reported that cash transfer had no effect on HIV prevalence in South Africa among in-school adolescent girls (adjusted odds ratio [OR] 1.17, 95% CI 0.80-1.72) [29] and among men and women in Malawi conditional on remaining HIV-negative (β=.001, robust SE=.005) [30]. Cash transfer studies among in-school adolescent orphans showed no effect on HIV infection (OR 1.15, 95% CI 0.47-2.79) and HSV-2 infection (OR 1.46, 95% CI 0.50-4.26) in Zimbabwe [31] and on HIV infection (adjusted OR 0.72, 95% CI 0.15-3.42) and HSV-2 infection (OR 0.98, 95% CI 0.54-4.26) in Kenya [32]. However, none of these studies in sub-Saharan Africa were conducted among out-of-school AGYW aged 15-23 years to ascertain the combined effect of cash transfer and combination HIV prevention (CHP) services".

2b) In INTRODUCTION: Specific objectives or hypotheses

Does your paper address CONSORT subitem 2b? *

Copy and paste relevant sections from the manuscript (include quotes in quotation marks "like this" to indicate direct quotes from your manuscript), or elaborate on this item by providing additional information not in the ms, or briefly explain why the item is not applicable/relevant for your study

"An impact evaluation was conducted to evaluate the efficacy of cash transfer delivered along with combination HIV prevention (CHP) services, on reduction of risky sexual behavior (CARE study) among AGYW in Shinyanga Region, Tanzania, where the Sauti (meaning Voices in Kiswahili) Project provided CHP services to AGYW under the DREAMS (Determined, Resilient, Empowered, AIDS-free, Mentored, and Safe) initiative in Tanzania"

METHODS

3a) Description of trial design (such as parallel, factorial) including allocation ratio

Does your paper address CONSORT subitem 3a? *

Copy and paste relevant sections from the manuscript (include quotes in quotation marks "like this" to indicate direct quotes from your manuscript), or elaborate on this item by providing additional information not in the ms, or briefly explain why the item is not applicable/relevant for your study

"This study was a 2-arm cluster-randomized controlled trial with 1:1 allocation ratio, implemented among out-of-school AGYW in Tanzania by the Sauti project—a project implementing community-based interventions under the DREAMS (Determined, Resilient, Empowered, AIDS-free, Mentored, and Safe) initiative in Tanzania [4,33]."

3b) Important changes to methods after trial commencement (such as eligibility criteria), with reasons

Does your paper address CONSORT subitem 3b? *

Copy and paste relevant sections from the manuscript (include quotes in quotation marks "like this" to indicate direct quotes from your manuscript), or elaborate on this item by providing additional information not in the ms, or briefly explain why the item is not applicable/relevant for your study

No changes were made to the study after trial commencement.

3b-i) Bug fixes, Downtimes, Content Changes

Bug fixes, Downtimes, Content Changes: ehealth systems are often dynamic systems. A description of changes to methods therefore also includes important changes made on the intervention or comparator during the trial (e.g., major bug fixes or changes in the functionality or content) (5-iii) and other "unexpected events" that may have influenced study design such as staff changes, system failures/downtimes, etc. [2].

	1	2	3	4	5	
subitem not at all important	(•)	( )	( )	( )	( )	essential

Clear selection

Does your paper address subitem 3b-i?

Copy and paste relevant sections from the manuscript (include quotes in quotation marks "like this" to indicate direct quotes from your manuscript), or elaborate on this item by providing additional information not in the ms, or briefly explain why the item is not applicable/relevant for your study

Your answer

4a) Eligibility criteria for participants

Does your paper address CONSORT subitem 4a? *

Copy and paste relevant sections from the manuscript (include quotes in quotation marks "like this" to indicate direct quotes from your manuscript), or elaborate on this item by providing additional information not in the ms, or briefly explain why the item is not applicable/relevant for your study

"AGYW were eligible for the study if they were aged 15-23 years; out-of-school (defined as either never enrolled or have dropped out of school for at least a month at the time of study enrolment) as documented by the household survey; residents of the village of recruitment; had completed 10 hours of SBCC training; willing to take part in the study, including testing for HIV and receiving results; and willing to participate in the cash transfer program (applicable to the intervention arm)"

4a-i) Computer / Internet literacy

Computer / Internet literacy is often an implicit “de facto” eligibility criterion - this should be explicitly clarified.

	1	2	3	4	5	
subitem not at all important	(selected)	○	○	○	○	essential

Clear selection

Does your paper address subitem 4a-i?

Copy and paste relevant sections from the manuscript (include quotes in quotation marks "like this" to indicate direct quotes from your manuscript), or elaborate on this item by providing additional information not in the ms, or briefly explain why the item is not applicable/relevant for your study

Your answer

4a-ii) Open vs. closed, web-based vs. face-to-face assessments:

Open vs. closed, web-based vs. face-to-face assessments: Mention how participants were recruited (online vs. offline), e.g., from an open access website or from a clinic, and clarify if this was a purely web-based trial, or there were face-to-face components (as part of the intervention or for assessment), i.e., to what degree got the study team to know the participant. In online-only trials, clarify if participants were quasi-anonymous and whether having multiple identities was possible or whether technical or logistical measures (e.g., cookies, email confirmation, phone calls) were used to detect/prevent these.

	1	2	3	4	5	
subitem not at all important	○	○	○	○	◉	essential

Clear selection

Does your paper address subitem 4a-ii? *

Copy and paste relevant sections from the manuscript (include quotes in quotation marks "like this" to indicate direct quotes from your manuscript), or elaborate on this item by providing additional information not in the ms, or briefly explain why the item is not applicable/relevant for your study

"The CARE Study enrolled participants from a pool of potential Sauti Project DREAMS beneficiaries in Shinyanga Region. Prior to the recruitment, Sauti project staff identified villages that were potentially eligible for the CT Program based on the local multidimensional poverty index and consultations with local stakeholders and experts from CSOs that worked with AGYW under the Sauti Project. Thereafter, the Project conducted a household survey among AGYW in the identified villages to determine AGYW who were out of-school and develop a pool of potential CTP beneficiaries. Sensitization meetings with these pools and their parents/guardians were conducted to introduce the Sauti project CHP (biomedical, behavioral and structural) interventions. Potential beneficiaries were then invited to attend ten sessions of social and behavior change communication (SBCC) and other project interventions. The SBCC sessions were peer-led group sessions designed to address significant determinants of HIV risk, gender and reproductive health. During the SBCC sessions, data on all villages receiving CHP was generated for CARE study eligibility assessment and randomization. In the identified CARE Study clusters (intervention and control), potential participants were also given information about the CARE study during group meetings for SBCC. After the 10 hours of SBCC sessions, AGYW were informed about CARE study enrolment, recruitment dates and locations."

4a-iii) Information giving during recruitment

Information given during recruitment. Specify how participants were briefed for recruitment and in the informed consent procedures (e.g., publish the informed consent documentation as appendix, see also item X26), as this information may have an effect on user self-selection, user expectation and may also bias results.

	1	2	3	4	5	
subitem not at all important	○	○	○	○	◉	essential

Clear selection

Does your paper address subitem 4a-iii?

Copy and paste relevant sections from the manuscript (include quotes in quotation marks "like this" to indicate direct quotes from your manuscript), or elaborate on this item by providing additional information not in the ms, or briefly explain why the item is not applicable/relevant for your study

"Each study participant provided written informed consent or assent. All girls younger than 18 years of age required consent from parents/guardian and their assent to participate in the study, except for emancipated minors (i.e. minors who are married, have given birth or demonstrating full independence, e.g., living alone or heading the household). Consent and assent forms were administered in Kiswahili."

4b) Settings and locations where the data were collected

Does your paper address CONSORT subitem 4b? *

Copy and paste relevant sections from the manuscript (include quotes in quotation marks "like this" to indicate direct quotes from your manuscript), or elaborate on this item by providing additional information not in the ms, or briefly explain why the item is not applicable/relevant for your study

"The study was conducted in three contiguous district councils: Kahama Town Council, Ushetu and Msalala District Councils in Shinyanga region.

4b-i) Report if outcomes were (self-)assessed through online questionnaires

Clearly report if outcomes were (self-)assessed through online questionnaires (as common in web-based trials) or otherwise.

	1	2	3	4	5	
subitem not at all important	(●)	( )	( )	( )	( )	essential

Clear selection

Does your paper address subitem 4b-i? *

Copy and paste relevant sections from the manuscript (include quotes in quotation marks "like this" to indicate direct quotes from your manuscript), or elaborate on this item by providing additional information not in the ms, or briefly explain why the item is not applicable/relevant for your study

No online questionnaire were used for this study

4b-ii) Report how institutional affiliations are displayed

Report how institutional affiliations are displayed to potential participants [on ehealth media], as affiliations with prestigious hospitals or universities may affect volunteer rates, use, and reactions with regards to an intervention.(Not a required item – describe only if this may bias results)

	1	2	3	4	5	
subitem not at all important	(•)	( )	( )	( )	( )	essential

Clear selection

Does your paper address subitem 4b-ii?

Copy and paste relevant sections from the manuscript (include quotes in quotation marks "like this" to indicate direct quotes from your manuscript), or elaborate on this item by providing additional information not in the ms, or briefly explain why the item is not applicable/relevant for your study

Your answer

5) The interventions for each group with sufficient details to allow replication, including how and when they were actually administered

5-i) Mention names, credential, affiliations of the developers, sponsors, and owners

Mention names, credential, affiliations of the developers, sponsors, and owners [6] (if authors/evaluators are owners or developer of the software, this needs to be declared in a "Conflict of interest" section or mentioned elsewhere in the manuscript).

	1	2	3	4	5	
subitem not at all important	( )	( )	( )	( )	(•)	essential

Clear selection

Does your paper address subitem 5-i?

Copy and paste relevant sections from the manuscript (include quotes in quotation marks "like this" to indicate direct quotes from your manuscript), or elaborate on this item by providing additional information not in the ms, or briefly explain why the item is not applicable/relevant for your study

Participants in the intervention arm received CHP and unconditional cash transfer in quarterly instalments of 70,000 Tanzania shillings (~US $31) for 18 months through mobile money on a project-provided cellular phone. Participants in the control arm received CHP only and cellular phones provided by the project. The aim was to make sure that the 2 arms were comparable except for the intervention. The interventions are described elsewhere [33]. In short, all study participants received Sauti's core package of interventions, including risk reduction counselling, HIV testing services, condom use skills and provision, family planning counselling and service provision, sexually transmitted infection screening and treatment, gender-based violence interventions (escorted referrals and the desk for social, legal, and medical services provided to survivors of gender-based violence), tuberculosis and alcohol and drug abuse screening, and referral to services. The other features were SBCC training sessions and economic empowerment community banking also called as the Women Organizing Resources Together plus (WORTH+) intervention [33]. The cash transfer program was implemented in the intervention arm only. The WORTH+ intervention consisted of financial literacy training that aimed to build microbusiness development skills and community banking.

5-ii) Describe the history/development process

Describe the history/development process of the application and previous formative evaluations (e.g., focus groups, usability testing), as these will have an impact on adoption/use rates and help with interpreting results.

	1	2	3	4	5	
subitem not at all important	(●)	( )	( )	( )	( )	essential

Clear selection

Does your paper address subitem 5-ii?

Copy and paste relevant sections from the manuscript (include quotes in quotation marks "like this" to indicate direct quotes from your manuscript), or elaborate on this item by providing additional information not in the ms, or briefly explain why the item is not applicable/relevant for your study

Your answer

5-iii) Revisions and updating

Revisions and updating. Clearly mention the date and/or version number of the application/intervention (and comparator, if applicable) evaluated, or describe whether the intervention underwent major changes during the evaluation process, or whether the development and/or content was “frozen” during the trial. Describe dynamic components such as news feeds or changing content which may have an impact on the replicability of the intervention (for unexpected events see item 3b).

	1	2	3	4	5	
subitem not at all important	(●)	( )	( )	( )	( )	essential

Clear selection

Does your paper address subitem 5-iii?

Copy and paste relevant sections from the manuscript (include quotes in quotation marks "like this" to indicate direct quotes from your manuscript), or elaborate on this item by providing additional information not in the ms, or briefly explain why the item is not applicable/relevant for your study

Your answer

5-iv) Quality assurance methods

Provide information on quality assurance methods to ensure accuracy and quality of information provided [1], if applicable.

	1	2	3	4	5	
subitem not at all important	○	○	○	◉	○	essential

Clear selection

Does your paper address subitem 5-iv?

Copy and paste relevant sections from the manuscript (include quotes in quotation marks "like this" to indicate direct quotes from your manuscript), or elaborate on this item by providing additional information not in the ms, or briefly explain why the item is not applicable/relevant for your study

"The study used ACASI to collect sexual behavior and other sensitive data. Studies comparing face to face interviews with ACASI have reported that respondents are more likely to be open and honest when using ACASI"

5-v) Ensure replicability by publishing the source code, and/or providing screenshots/screen-capture video, and/or providing flowcharts of the algorithms used

Ensure replicability by publishing the source code, and/or providing screenshots/screen-capture video, and/or providing flowcharts of the algorithms used. Replicability (i.e., other researchers should in principle be able to replicate the study) is a hallmark of scientific reporting.

	1	2	3	4	5	
subitem not at all important	○	○	○	○	◉	essential

Clear selection

Does your paper address subitem 5-v?

Copy and paste relevant sections from the manuscript (include quotes in quotation marks "like this" to indicate direct quotes from your manuscript), or elaborate on this item by providing additional information not in the ms, or briefly explain why the item is not applicable/relevant for your study

The intervention has been described in detail above

5-vi) Digital preservation

Digital preservation: Provide the URL of the application, but as the intervention is likely to change or disappear over the course of the years; also make sure the intervention is archived (Internet Archive, webcitation.org, and/or publishing the source code or screenshots/videos alongside the article). As pages behind login screens cannot be archived, consider creating demo pages which are accessible without login.

	1	2	3	4	5	
subitem not at all important	(●)	( )	( )	( )	( )	essential

Clear selection

Does your paper address subitem 5-vi?

Copy and paste relevant sections from the manuscript (include quotes in quotation marks "like this" to indicate direct quotes from your manuscript), or elaborate on this item by providing additional information not in the ms, or briefly explain why the item is not applicable/relevant for your study

Your answer

5-vii) Access

Access: Describe how participants accessed the application, in what setting/context, if they had to pay (or were paid) or not, whether they had to be a member of specific group. If known, describe how participants obtained “access to the platform and Internet” [1]. To ensure access for editors/reviewers/readers, consider to provide a “backdoor” login account or demo mode for reviewers/readers to explore the application (also important for archiving purposes, see vi).

	1	2	3	4	5	
subitem not at all important	(●)	( )	( )	( )	( )	essential

Clear selection

Does your paper address subitem 5-vii? *

Copy and paste relevant sections from the manuscript (include quotes in quotation marks "like this" to indicate direct quotes from your manuscript), or elaborate on this item by providing additional information not in the ms, or briefly explain why the item is not applicable/relevant for your study

No application was used in the current study

5-viii) Mode of delivery, features/functionalities/components of the intervention and comparator, and the theoretical framework

Describe mode of delivery, features/functionalities/components of the intervention and comparator, and the theoretical framework [6] used to design them (instructional strategy [1], behaviour change techniques, persuasive features, etc., see e.g., [7, 8] for terminology). This includes an in-depth description of the content (including where it is coming from and who developed it) [1],” whether [and how] it is tailored to individual circumstances and allows users to track their progress and receive feedback” [6]. This also includes a description of communication delivery channels and – if computer-mediated communication is a component – whether communication was synchronous or asynchronous [6]. It also includes information on presentation strategies [1], including page design principles, average amount of text on pages, presence of hyperlinks to other resources, etc. [1].

	1	2	3	4	5	
subitem not at all important	○	○	○	◉	○	essential

Clear selection

Does your paper address subitem 5-viii? *

Copy and paste relevant sections from the manuscript (include quotes in quotation marks "like this" to indicate direct quotes from your manuscript), or elaborate on this item by providing additional information not in the ms, or briefly explain why the item is not applicable/relevant for your study

Participants in the intervention arm received CHP and unconditional cash transfer in quarterly instalments of 70,000 Tanzania shillings (~US $31) for 18 months through mobile money on a project-provided cellular phone. Participants in the control arm received CHP only and cellular phones provided by the project. The aim was to make sure that the 2 arms were comparable except for the intervention. The interventions are described elsewhere [33]. In short, all study participants received Sauti's core package of interventions, including risk reduction counselling, HIV testing services, condom use skills and provision, family planning counselling and service provision, sexually transmitted infection screening and treatment, gender-based violence interventions (escorted referrals and the desk for social, legal, and medical services provided to survivors of gender-based violence), tuberculosis and alcohol and drug abuse screening, and referral to services. The other features were SBCC training sessions and economic empowerment community banking also called as the Women Organizing Resources Together plus (WORTH+) intervention [33]. The cash transfer program was implemented in the intervention arm only. The WORTH+ intervention consisted of financial literacy training that aimed to build microbusiness development skills and community banking.

5-ix) Describe use parameters

Describe use parameters (e.g., intended "doses" and optimal timing for use). Clarify what instructions or recommendations were given to the user, e.g., regarding timing, frequency, heaviness of use, if any, or was the intervention used ad libitum.

	1	2	3	4	5	
subitem not at all important	○	○	○	○	●	essential

Clear selection

Does your paper address subitem 5-ix?

Copy and paste relevant sections from the manuscript (include quotes in quotation marks "like this" to indicate direct quotes from your manuscript), or elaborate on this item by providing additional information not in the ms, or briefly explain why the item is not applicable/relevant for your study

The see the above response

5-x) Clarify the level of human involvement

Clarify the level of human involvement (care providers or health professionals, also technical assistance) in the e-intervention or as co-intervention (detail number and expertise of professionals involved, if any, as well as “type of assistance offered, the timing and frequency of the support, how it is initiated, and the medium by which the assistance is delivered”. It may be necessary to distinguish between the level of human involvement required for the trial, and the level of human involvement required for a routine application outside of a RCT setting (discuss under item 21 – generalizability).

	1	2	3	4	5	
subitem not at all important	○	○	○	○	◉	essential

Clear selection

Does your paper address subitem 5-x?

Copy and paste relevant sections from the manuscript (include quotes in quotation marks "like this" to indicate direct quotes from your manuscript), or elaborate on this item by providing additional information not in the ms, or briefly explain why the item is not applicable/relevant for your study

"Trained government healthcare workers offered HIV pre-test counselling, blood draw for HIV and HSV-2 testing to the participants as per the National Guideline for the Management of HIV and AIDS".

5-xi) Report any prompts/reminders used

Report any prompts/reminders used: Clarify if there were prompts (letters, emails, phone calls, SMS) to use the application, what triggered them, frequency etc. It may be necessary to distinguish between the level of prompts/reminders required for the trial, and the level of prompts/reminders for a routine application outside of a RCT setting (discuss under item 21 – generalizability).

	1	2	3	4	5	
subitem not at all important	(●)	( )	( )	( )	( )	essential

Clear selection

Does your paper address subitem 5-xi? *

Copy and paste relevant sections from the manuscript (include quotes in quotation marks "like this" to indicate direct quotes from your manuscript), or elaborate on this item by providing additional information not in the ms, or briefly explain why the item is not applicable/relevant for your study

No reminders were used for this study

5-xii) Describe any co-interventions (incl. training/support)

Describe any co-interventions (incl. training/support): Clearly state any interventions that are provided in addition to the targeted eHealth intervention, as ehealth intervention may not be designed as stand-alone intervention. This includes training sessions and support [1]. It may be necessary to distinguish between the level of training required for the trial, and the level of training for a routine application outside of a RCT setting (discuss under item 21 – generalizability.

	1	2	3	4	5	
subitem not at all important	(●)	( )	( )	( )	( )	essential

Clear selection

Does your paper address subitem 5-xii? *

Copy and paste relevant sections from the manuscript (include quotes in quotation marks "like this" to indicate direct quotes from your manuscript), or elaborate on this item by providing additional information not in the ms, or briefly explain why the item is not applicable/relevant for your study

No co-intervention existed for this study

6a) Completely defined pre-specified primary and secondary outcome measures, including how and when they were assessed

Does your paper address CONSORT subitem 6a? *

Copy and paste relevant sections from the manuscript (include quotes in quotation marks "like this" to indicate direct quotes from your manuscript), or elaborate on this item by providing additional information not in the ms, or briefly explain why the item is not applicable/relevant for your study

"The primary outcome was HSV-2 incidence, which was established at each follow-up based on the baseline HSV-2 infection. Secondary outcomes included: HIV prevalence at follow-up, self-reported intergenerational sex and self-reported compensated sex. All secondary outcomes were measured at baseline and each study follow-up visit."

6a-i) Online questionnaires: describe if they were validated for online use and apply CHERRIES items to describe how the questionnaires were designed/deployed

If outcomes were obtained through online questionnaires, describe if they were validated for online use and apply CHERRIES items to describe how the questionnaires were designed/deployed [9].

	1	2	3	4	5	
subitem not at all important	◉	○	○	○	○	essential

Clear selection

Does your paper address subitem 6a-i?

Copy and paste relevant sections from manuscript text

Your answer

6a-ii) Describe whether and how “use” (including intensity of use/dosage) was defined/measured/monitored

Describe whether and how “use” (including intensity of use/dosage) was defined/measured/monitored (logins, logfile analysis, etc.). Use/adoption metrics are important process outcomes that should be reported in any ehealth trial.

	1	2	3	4	5	
subitem not at all important	◉	○	○	○	○	essential

Clear selection

Does your paper address subitem 6a-ii?

Copy and paste relevant sections from manuscript text

Your answer

6a-iii) Describe whether, how, and when qualitative feedback from participants was obtained

Describe whether, how, and when qualitative feedback from participants was obtained (e.g., through emails, feedback forms, interviews, focus groups).

	1	2	3	4	5	
subitem not at all important	◉	○	○	○	○	essential

Clear selection

Does your paper address subitem 6a-iii?

Copy and paste relevant sections from manuscript text

Your answer

6b) Any changes to trial outcomes after the trial commenced, with reasons

Does your paper address CONSORT subitem 6b? *

Copy and paste relevant sections from the manuscript (include quotes in quotation marks "like this" to indicate direct quotes from your manuscript), or elaborate on this item by providing additional information not in the ms, or briefly explain why the item is not applicable/relevant for your study

No changes were made to trial outcomes

7a) How sample size was determined

NPT: When applicable, details of whether and how the clustering by care provides or centers was addressed

7a-i) Describe whether and how expected attrition was taken into account when calculating the sample size

Describe whether and how expected attrition was taken into account when calculating the sample size.

	1	2	3	4	5	
subitem not at all important	○	○	○	○	◉	essential

Clear selection

Does your paper address subitem 7a-i?

Copy and paste relevant sections from manuscript title (include quotes in quotation marks "like this" to indicate direct quotes from your manuscript), or elaborate on this item by providing additional information not in the ms, or briefly explain why the item is not applicable/relevant for your study

"All calculations for sample size were conducted using methods for matched cluster randomized trials [38]. A sample of 14 paired clusters (28 clusters) with 70 participants per cluster was estimated to achieve more than 80% power of detecting a 35% reduction in HSV2 incidence in the intervention arm at the end of 18-month period."

7b) When applicable, explanation of any interim analyses and stopping guidelines

Does your paper address CONSORT subitem 7b? *

Copy and paste relevant sections from the manuscript (include quotes in quotation marks "like this" to indicate direct quotes from your manuscript), or elaborate on this item by providing additional information not in the ms, or briefly explain why the item is not applicable/relevant for your study

No interim analysis was conducted for this study

8a) Method used to generate the random allocation sequence
NPT: When applicable, how care providers were allocated to each trial group

Does your paper address CONSORT subitem 8a? *

Copy and paste relevant sections from the manuscript (include quotes in quotation marks "like this" to indicate direct quotes from your manuscript), or elaborate on this item by providing additional information not in the ms, or briefly explain why the item is not applicable/relevant for your study

This is not applicable to the current study, as it was a cluster randomized trial

8b) Type of randomisation; details of any restriction (such as blocking and block size)

Does your paper address CONSORT subitem 8b? *

Copy and paste relevant sections from the manuscript (include quotes in quotation marks "like this" to indicate direct quotes from your manuscript), or elaborate on this item by providing additional information not in the ms, or briefly explain why the item is not applicable/relevant for your study

"Villages (Clusters) were the unit of randomization for this study"

9) Mechanism used to implement the random allocation sequence (such as sequentially numbered containers), describing any steps taken to conceal the sequence until interventions were assigned

Does your paper address CONSORT subitem 9? *

Copy and paste relevant sections from the manuscript (include quotes in quotation marks "like this" to indicate direct quotes from your manuscript), or elaborate on this item by providing additional information not in the ms, or briefly explain why the item is not applicable/relevant for your study

This was not applicable to this study

10) Who generated the random allocation sequence, who enrolled participants, and who assigned participants to interventions

Does your paper address CONSORT subitem 10? *

Copy and paste relevant sections from the manuscript (include quotes in quotation marks "like this" to indicate direct quotes from your manuscript), or elaborate on this item by providing additional information not in the ms, or briefly explain why the item is not applicable/relevant for your study

This was not applicable to this study

11a) If done, who was blinded after assignment to interventions (for example, participants, care providers, those assessing outcomes) and how

NPT: Whether or not administering co-interventions were blinded to group assignment

11a-i) Specify who was blinded, and who wasn’t

Specify who was blinded, and who wasn’t. Usually, in web-based trials it is not possible to blind the participants [1, 3] (this should be clearly acknowledged), but it may be possible to blind outcome assessors, those doing data analysis or those administering co-interventions (if any).

	1	2	3	4	5	
subitem not at all important	○	○	◉	○	○	essential

Clear selection

Does your paper address subitem 11a-i? *

Copy and paste relevant sections from the manuscript (include quotes in quotation marks "like this" to indicate direct quotes from your manuscript), or elaborate on this item by providing additional information not in the ms, or briefly explain why the item is not applicable/relevant for your study

o blinding was performed in this study.

11a-ii) Discuss e.g., whether participants knew which intervention was the “intervention of interest” and which one was the “comparator”

Informed consent procedures (4a-ii) can create biases and certain expectations - discuss e.g., whether participants knew which intervention was the “intervention of interest” and which one was the “comparator”.

	1	2	3	4	5	
subitem not at all important	○	○	○	○	◉	essential

Clear selection

Does your paper address subitem 11a-ii?

Copy and paste relevant sections from the manuscript (include quotes in quotation marks "like this" to indicate direct quotes from your manuscript), or elaborate on this item by providing additional information not in the ms, or briefly explain why the item is not applicable/relevant for your study

"Each study participant provided written informed consent or assent. All girls younger than 18 years of age required consent from parents/guardian and their assent to participate in the study, except for emancipated minors (i.e. minors who are married, have given birth or demonstrating full independence, e.g., living alone or heading the household)."
No blinding was performed in this study because it was not possible to blind study participants, Sauti implementers, data collectors, and data analysts as the intervention provided (cash transfer) was known to study participants, implementers, and researchers.

11b) If relevant, description of the similarity of interventions

(this item is usually not relevant for ehealth trials as it refers to similarity of a placebo or sham intervention to a active medication/intervention)

Does your paper address CONSORT subitem 11b? *

Copy and paste relevant sections from the manuscript (include quotes in quotation marks "like this" to indicate direct quotes from your manuscript), or elaborate on this item by providing additional information not in the ms, or briefly explain why the item is not applicable/relevant for your study

This is not applicable to the current study

12a) Statistical methods used to compare groups for primary and secondary outcomes

NPT: When applicable, details of whether and how the clustering by care providers or centers was addressed

Does your paper address CONSORT subitem 12a? *

Copy and paste relevant sections from the manuscript (include quotes in quotation marks "like this" to indicate direct quotes from your manuscript), or elaborate on this item by providing additional information not in the ms, or briefly explain why the item is not applicable/relevant for your study

"All eligible clusters were matched into pairs by location (rural vs. urban areas) and the presence or absence of HIV high-risk areas generating three strata; rural clusters in the high-risk area, urban clusters, rural clusters in the low-risk area. Matching was conducted to minimize the between-community variance in HSV-2 incidence within the matched clusters. From each matched pair, one cluster was randomly selected to receive the intervention package and the other automatically assigned to the control arm."

12a-i) Imputation techniques to deal with attrition / missing values

Imputation techniques to deal with attrition / missing values: Not all participants will use the intervention/comparator as intended and attrition is typically high in ehealth trials. Specify how participants who did not use the application or dropped out from the trial were treated in the statistical analysis (a complete case analysis is strongly discouraged, and simple imputation techniques such as LOCF may also be problematic [4]).

	1	2	3	4	5	
subitem not at all important	○	○	○	◉	○	essential

Clear selection

Does your paper address subitem 12a-i? *

Copy and paste relevant sections from the manuscript (include quotes in quotation marks "like this" to indicate direct quotes from your manuscript), or elaborate on this item by providing additional information not in the ms, or briefly explain why the item is not applicable/relevant for your study

No imputation techniques were done to deal with attrition in the current study

12b) Methods for additional analyses, such as subgroup analyses and adjusted analyses

Does your paper address CONSORT subitem 12b? *

Copy and paste relevant sections from the manuscript (include quotes in quotation marks "like this" to indicate direct quotes from your manuscript), or elaborate on this item by providing additional information not in the ms, or briefly explain why the item is not applicable/relevant for your study

"Secondary analysis of the effect of cash transfer on HIV prevalence at follow-up was estimated using a log-binomial model adjusting for matched pairs, age, and other variables with baseline imbalance between the arms and adjusting for standard errors for clustering at the village level. Intergenerational sex, compensated sex, transactional sex, and other behavioral outcomes were compared between study arms by using generalized estimating equations with identity logit, binomial distribution, and robust variance to account for repeated measures on each participant. The generalized estimating equations regression models were also fitted to assess for interaction between the trial arm and strata (rural cluster in the high-risk area, urban cluster, rural cluster in the low-risk area)".

X26) REB/IRB Approval and Ethical Considerations [recommended as subheading under "Methods"] (not a CONSORT item)

X26-i) Comment on ethics committee approval

	1	2	3	4	5	
subitem not at all important	○	○	○	○	◉	essential

Clear selection

Does your paper address subitem X26-i?

Copy and paste relevant sections from the manuscript (include quotes in quotation marks "like this" to indicate direct quotes from your manuscript), or elaborate on this item by providing additional information not in the ms, or briefly explain why the item is not applicable/relevant for your study

"Ethical approval for this study was obtained from the John Hopkins University School of Public Health Research Ethics Committee (00007976) and the Tanzanian Medical Research Coordinating Committee of the National Institute for Medical Research (NIMR) (NIMR/HQ/R.8a/Vol.IX/2287)."

x26-ii) Outline informed consent procedures

Outline informed consent procedures e.g., if consent was obtained offline or online (how? Checkbox, etc.?), and what information was provided (see 4a-ii). See [6] for some items to be included in informed consent documents.

	1	2	3	4	5	
subitem not at all important	○	○	○	○	◉	essential

Clear selection

Does your paper address subitem X26-ii?

Copy and paste relevant sections from the manuscript (include quotes in quotation marks "like this" to indicate direct quotes from your manuscript), or elaborate on this item by providing additional information not in the ms, or briefly explain why the item is not applicable/relevant for your study

"Each study participant provided written informed consent or assent. "

X26-iii) Safety and security procedures

Safety and security procedures, incl. privacy considerations, and any steps taken to reduce the likelihood or detection of harm (e.g., education and training, availability of a hotline)

	1	2	3	4	5	
subitem not at all important	○	○	○	○	◉	essential

Clear selection

Does your paper address subitem X26-iii?

Copy and paste relevant sections from the manuscript (include quotes in quotation marks "like this" to indicate direct quotes from your manuscript), or elaborate on this item by providing additional information not in the ms, or briefly explain why the item is not applicable/relevant for your study

"All study procedures were conducted in a confidential environment in pre-identified venues in the respective communities"

RESULTS

13a) For each group, the numbers of participants who were randomly assigned, received intended treatment, and were analysed for the primary outcome

NPT: The number of care providers or centers performing the intervention in each group and the number of patients treated by each care provider in each center

Does your paper address CONSORT subitem 13a? *

Copy and paste relevant sections from the manuscript (include quotes in quotation marks "like this" to indicate direct quotes from your manuscript), or elaborate on this item by providing additional information not in the ms, or briefly explain why the item is not applicable/relevant for your study

"3071 were eligible and 3055 consented for study participation. Of those who consented, 3026 (1482 intervention, 1544 control) participants completed baseline survey procedures"

13b) For each group, losses and exclusions after randomisation, together with reasons

Does your paper address CONSORT subitem 13b? (NOTE: Preferably, this is shown in a CONSORT flow diagram) *

Copy and paste relevant sections from the manuscript (include quotes in quotation marks "like this" to indicate direct quotes from your manuscript), or elaborate on this item by providing additional information not in the ms, or briefly explain why the item is not applicable/relevant for your study

"Of the 3105 participants screened for eligibility (Figure 1), 3071 were eligible and 3055 consented for study participation. Of those who consented, 3026 (1482 intervention, 1544 control) participants completed baseline survey procedures and 2720 (1373 intervention, 1347 control) attended at least one follow-up study visit. Of those with follow-up data, 865 (443 intervention, 422 control) were infected with HSV-2 at baseline and therefore excluded in the HSV-2 longitudinal analysis." A flow diagram is also included in the manuscript to elaborate on this.

13b-i) Attrition diagram

Strongly recommended: An attrition diagram (e.g., proportion of participants still logging in or using the intervention/comparator in each group plotted over time, similar to a survival curve) or other figures or tables demonstrating usage/dose/engagement.

	1	2	3	4	5	
subitem not at all important	(●)	( )	( )	( )	( )	essential

Clear selection

Does your paper address subitem 13b-i?

Copy and paste relevant sections from the manuscript or cite the figure number if applicable (include quotes in quotation marks "like this" to indicate direct quotes from your manuscript), or elaborate on this item by providing additional information not in the ms, or briefly explain why the item is not applicable/relevant for your study

Your answer

14a) Dates defining the periods of recruitment and follow-up

Does your paper address CONSORT subitem 14a? *

Copy and paste relevant sections from the manuscript (include quotes in quotation marks "like this" to indicate direct quotes from your manuscript), or elaborate on this item by providing additional information not in the ms, or briefly explain why the item is not applicable/relevant for your study

Study recruitment took place between October, 30th and December 1, 2017."

14a-i) Indicate if critical “secular events” fell into the study period

Indicate if critical “secular events” fell into the study period, e.g., significant changes in Internet resources available or “changes in computer hardware or Internet delivery resources”

	1	2	3	4	5	
subitem not at all important	(●)	( )	( )	( )	( )	essential

Clear selection

Does your paper address subitem 14a-i?

Copy and paste relevant sections from the manuscript (include quotes in quotation marks "like this" to indicate direct quotes from your manuscript), or elaborate on this item by providing additional information not in the ms, or briefly explain why the item is not applicable/relevant for your study

Your answer

14b) Why the trial ended or was stopped (early)

Does your paper address CONSORT subitem 14b? *

Copy and paste relevant sections from the manuscript (include quotes in quotation marks "like this" to indicate direct quotes from your manuscript), or elaborate on this item by providing additional information not in the ms, or briefly explain why the item is not applicable/relevant for your study

The trial ended as planned

15) A table showing baseline demographic and clinical characteristics for each group

NPT: When applicable, a description of care providers (case volume, qualification, expertise, etc.) and centers (volume) in each group

Does your paper address CONSORT subitem 15? *

Copy and paste relevant sections from the manuscript (include quotes in quotation marks "like this" to indicate direct quotes from your manuscript), or elaborate on this item by providing additional information not in the ms, or briefly explain why the item is not applicable/relevant for your study

"At baseline, the median age of participants was 20 years (IQR 18-22 years) and the intervention and control groups were similar for key sexual behavior variables, HIV and HSV-2 prevalence. However, there were imbalances on socio-demographic variables such as age, marital status, debt and going to bed hungry"

15-i) Report demographics associated with digital divide issues

In ehealth trials it is particularly important to report demographics associated with digital divide issues, such as age, education, gender, social-economic status, computer/Internet/ehealth literacy of the participants, if known.

	1	2	3	4	5	
subitem not at all important	(●)	( )	( )	( )	( )	essential

Clear selection

Does your paper address subitem 15-i? *

Copy and paste relevant sections from the manuscript (include quotes in quotation marks "like this" to indicate direct quotes from your manuscript), or elaborate on this item by providing additional information not in the ms, or briefly explain why the item is not applicable/relevant for your study

This is not applicable to the current study

16) For each group, number of participants (denominator) included in each analysis and whether the analysis was by original assigned groups

16-i) Report multiple “denominators” and provide definitions

Report multiple “denominators” and provide definitions: Report N’s (and effect sizes) “across a range of study participation [and use] thresholds” [1], e.g., N exposed, N consented, N used more than x times, N used more than y weeks, N participants “used” the intervention/comparator at specific pre-defined time points of interest (in absolute and relative numbers per group). Always clearly define “use” of the intervention.

	1	2	3	4	5	
subitem not at all important	○	○	○	○	◉	essential

Clear selection

Does your paper address subitem 16-i? *

Copy and paste relevant sections from the manuscript (include quotes in quotation marks "like this" to indicate direct quotes from your manuscript), or elaborate on this item by providing additional information not in the ms, or briefly explain why the item is not applicable/relevant for your study

"Of those who consented, 3026 (1482 intervention, 1544 control) participants completed baseline survey procedures and 2720 (1373 intervention, 1347 control) attended at least one follow-up study visit."
"Of the 1373 AGYW in the intervention arm included in the longitudinal data analysis, 465 (33.9%), 373 (27.2%), and 414 (30.2%) received cash transfer 3 times or less, 4 times, and over 4 times, respectively, while 121 (8.8%) did not disclose the number of times they received cash transfer"

16-ii) Primary analysis should be intent-to-treat

Primary analysis should be intent-to-treat, secondary analyses could include comparing only “users”, with the appropriate caveats that this is no longer a randomized sample (see 18-i).

	1	2	3	4	5	
subitem not at all important	○	○	○	○	◉	essential

Clear selection

Does your paper address subitem 16-ii?

Copy and paste relevant sections from the manuscript (include quotes in quotation marks "like this" to indicate direct quotes from your manuscript), or elaborate on this item by providing additional information not in the ms, or briefly explain why the item is not applicable/relevant for your study

All analyses performed were prespecified. The primary analysis was intention-to-treat and based on individual-level data because the study had a sufficient number of clusters per arm, and the cluster size was anticipated to differ considerably at follow-up

17a) For each primary and secondary outcome, results for each group, and the estimated effect size and its precision (such as 95% confidence interval)

Does your paper address CONSORT subitem 17a? *

Copy and paste relevant sections from the manuscript (include quotes in quotation marks "like this" to indicate direct quotes from your manuscript), or elaborate on this item by providing additional information not in the ms, or briefly explain why the item is not applicable/relevant for your study

Two hundred incident HSV-2 infections were diagnosed in the study (98 in the control and 102 in the intervention), resulting in an annual incidence of 7.9/100 (95% CI 6.9/100-9.1/100) PYO. There was no significant difference in HSV-2 incidence between the study arms (adjusted hazard ratio 0.96, 95% CI 0.67-1.38; P=.83; Table 2), although location significantly modified the effect of cash transfer on HSV-2 incidence (effect modification P<.001). In urban and rural areas at high risk of HIV infection, cash transfer was associated with a nonsignificant increased hazard ratio adjusted for baseline confounders (Multimedia Appendix 1 and Multimedia Appendix 2). However, in rural areas with a low risk of HIV infection, the adjusted hazard ratio for incident HSV-2 infection in the cash transfer group was 0.45 (95% CI 0.29-0.71; P=.001; Multimedia Appendix 3).

17a-i) Presentation of process outcomes such as metrics of use and intensity of use

In addition to primary/secondary (clinical) outcomes, the presentation of process outcomes such as metrics of use and intensity of use (dose, exposure) and their operational definitions is critical. This does not only refer to metrics of attrition (13-b) (often a binary variable), but also to more continuous exposure metrics such as “average session length”. These must be accompanied by a technical description how a metric like a “session” is defined (e.g., timeout after idle time) [1] (report under item 6a).

	1	2	3	4	5	
subitem not at all important	○	○	○	◉	○	essential

Clear selection

Does your paper address subitem 17a-i?

Copy and paste relevant sections from the manuscript (include quotes in quotation marks "like this" to indicate direct quotes from your manuscript), or elaborate on this item by providing additional information not in the ms, or briefly explain why the item is not applicable/relevant for your study

""CT intervention was successfully provided in all 15 intervention communities. Of the 1373 AGYW in the intervention arm included in the longitudinal data analysis, 465 (33.9%), 373 (27.2%), and 414 (30.2%) received cash transfer 3 times or less, 4 times, and over 4 times, respectively, while 121 (8.8%) did not disclose the number of times they received cash transfer"

17b) For binary outcomes, presentation of both absolute and relative effect sizes is recommended

Does your paper address CONSORT subitem 17b? *

Copy and paste relevant sections from the manuscript (include quotes in quotation marks "like this" to indicate direct quotes from your manuscript), or elaborate on this item by providing additional information not in the ms, or briefly explain why the item is not applicable/relevant for your study

This is included in the results reported on the table

18) Results of any other analyses performed, including subgroup analyses and adjusted analyses, distinguishing pre-specified from exploratory

Does your paper address CONSORT subitem 18? *

Copy and paste relevant sections from the manuscript (include quotes in quotation marks "like this" to indicate direct quotes from your manuscript), or elaborate on this item by providing additional information not in the ms, or briefly explain why the item is not applicable/relevant for your study

Overall, HSV-2 incidence was not significantly different at all follow-up points between the study arms in the adjusted analysis (hazard ratio 0.96, 95% CI 0.67-1.38; P=.83). However, HSV-2 incidence was significantly lower in the rural low-risk populations who received the cash transfer intervention (hazard ratio 0.45, 95% CI 0.29-0.71; P=.001), adjusted for potential confounders.

18-i) Subgroup analysis of comparing only users

A subgroup analysis of comparing only users is not uncommon in ehealth trials, but if done, it must be stressed that this is a self-selected sample and no longer an unbiased sample from a randomized trial (see 16-iii).

	1	2	3	4	5	
subitem not at all important	○	○	○	○	◉	essential

Clear selection

Does your paper address subitem 18-i?

Copy and paste relevant sections from the manuscript (include quotes in quotation marks "like this" to indicate direct quotes from your manuscript), or elaborate on this item by providing additional information not in the ms, or briefly explain why the item is not applicable/relevant for your study

Overall, HSV-2 incidence was not significantly different at all follow-up points between the study arms in the adjusted analysis (hazard ratio 0.96, 95% CI 0.67-1.38; P=.83). However, HSV-2 incidence was significantly lower in the rural low-risk populations who received the cash transfer intervention (hazard ratio 0.45, 95% CI 0.29-0.71; P=.001), adjusted for potential confounders.

19) All important harms or unintended effects in each group
(for specific guidance see CONSORT for harms)

Does your paper address CONSORT subitem 19? *

Copy and paste relevant sections from the manuscript (include quotes in quotation marks "like this" to indicate direct quotes from your manuscript), or elaborate on this item by providing additional information not in the ms, or briefly explain why the item is not applicable/relevant for your study

Only 3 social harm events were reported during the study—3 in the intervention and 0 in the control clusters—and were associated with minor teasing that cash transfer and phones provided to facilitate cash transfer may be associated with Freemasonry.

19-i) Include privacy breaches, technical problems

Include privacy breaches, technical problems. This does not only include physical “harm” to participants, but also incidents such as perceived or real privacy breaches [1], technical problems, and other unexpected/unintended incidents. “Unintended effects” also includes unintended positive effects [2].

	1	2	3	4	5	
subitem not at all important	(●)	( )	( )	( )	( )	essential

Clear selection

Does your paper address subitem 19-i?

Copy and paste relevant sections from the manuscript (include quotes in quotation marks "like this" to indicate direct quotes from your manuscript), or elaborate on this item by providing additional information not in the ms, or briefly explain why the item is not applicable/relevant for your study

Your answer

19-ii) Include qualitative feedback from participants or observations from staff/researchers

Include qualitative feedback from participants or observations from staff/researchers, if available, on strengths and shortcomings of the application, especially if they point to unintended/unexpected effects or uses. This includes (if available) reasons for why people did or did not use the application as intended by the developers.

	1	2	3	4	5	
subitem not at all important	(selected)	○	○	○	○	essential

Clear selection

Does your paper address subitem 19-ii?

Copy and paste relevant sections from the manuscript (include quotes in quotation marks "like this" to indicate direct quotes from your manuscript), or elaborate on this item by providing additional information not in the ms, or briefly explain why the item is not applicable/relevant for your study

Your answer

DISCUSSION

22) Interpretation consistent with results, balancing benefits and harms, and considering other relevant evidence

NPT: In addition, take into account the choice of the comparator, lack of or partial blinding, and unequal expertise of care providers or centers in each group

22-i) Restate study questions and summarize the answers suggested by the data, starting with primary outcomes and process outcomes (use)

Restate study questions and summarize the answers suggested by the data, starting with primary outcomes and process outcomes (use).

	1	2	3	4	5	
subitem not at all important	○	○	○	○	◉	essential

Clear selection

Does your paper address subitem 22-i? *

Copy and paste relevant sections from the manuscript (include quotes in quotation marks "like this" to indicate direct quotes from your manuscript), or elaborate on this item by providing additional information not in the ms, or briefly explain why the item is not applicable/relevant for your study

Overall, this study observed no significant effect of the quarterly cash transfer on HSV-2 incidence among out-of-school AGYW after 18 months of intervention. However, cash transfer was associated with a reduced incidence of HSV-2 infection in rural communities at low risk of HIV infections but not in urban and rural communities with high risk of HIV infections

22-ii) Highlight unanswered new questions, suggest future research

Highlight unanswered new questions, suggest future research.

	1	2	3	4	5	
subitem not at all important	○	○	○	○	◉	essential

Clear selection

Does your paper address subitem 22-ii?

Copy and paste relevant sections from the manuscript (include quotes in quotation marks "like this" to indicate direct quotes from your manuscript), or elaborate on this item by providing additional information not in the ms, or briefly explain why the item is not applicable/relevant for your study

Overall, this study observed no significant effect of the quarterly cash transfer on HSV-2 incidence among out-of-school AGYW after 18 months of intervention. However, cash transfer was associated with a reduced incidence of HSV-2 infection in rural communities at low risk of HIV infections but not in urban and rural communities with high risk of HIV infections

20) Trial limitations, addressing sources of potential bias, imprecision, and, if relevant, multiplicity of analyses

20-i) Typical limitations in ehealth trials

Typical limitations in ehealth trials: Participants in ehealth trials are rarely blinded. Ehealth trials often look at a multiplicity of outcomes, increasing risk for a Type I error. Discuss biases due to non-use of the intervention/usability issues, biases through informed consent procedures, unexpected events.

	1	2	3	4	5	
subitem not at all important	(●)	( )	( )	( )	( )	essential

Clear selection

Does your paper address subitem 20-i? *

Copy and paste relevant sections from the manuscript (include quotes in quotation marks "like this" to indicate direct quotes from your manuscript), or elaborate on this item by providing additional information not in the ms, or briefly explain why the item is not applicable/relevant for your study

The study had limitation. "Clusters were selected only from the Shinyanga Region, as the prevalence of HIV in this region was higher than the national average, and thus, these findings may not be generalizable to other study regions with lower HIV prevalence in Tanzania".

21) Generalisability (external validity, applicability) of the trial findings

NPT: External validity of the trial findings according to the intervention, comparators, patients, and care providers or centers involved in the trial

21-i) Generalizability to other populations

Generalizability to other populations: In particular, discuss generalizability to a general Internet population, outside of a RCT setting, and general patient population, including applicability of the study results for other organizations

	1	2	3	4	5	
subitem not at all important	○	○	○	○	◉	essential

Clear selection

Does your paper address subitem 21-i?

Copy and paste relevant sections from the manuscript (include quotes in quotation marks "like this" to indicate direct quotes from your manuscript), or elaborate on this item by providing additional information not in the ms, or briefly explain why the item is not applicable/relevant for your study

"This study has some limitations. The study clusters were selected only from the Shinyanga Region, as the prevalence of HIV in this region was higher than the national average, and thus, these findings may not be generalizable to other study regions with lower HIV prevalence in Tanzania.

21-ii) Discuss if there were elements in the RCT that would be different in a routine application setting

Discuss if there were elements in the RCT that would be different in a routine application setting (e.g., prompts/reminders, more human involvement, training sessions or other co-interventions) and what impact the omission of these elements could have on use, adoption, or outcomes if the intervention is applied outside of a RCT setting.

	1	2	3	4	5	
subitem not at all important	◉	○	○	○	○	essential

Clear selection

Does your paper address subitem 21-ii?

Copy and paste relevant sections from the manuscript (include quotes in quotation marks "like this" to indicate direct quotes from your manuscript), or elaborate on this item by providing additional information not in the ms, or briefly explain why the item is not applicable/relevant for your study

"The study clusters were selected from Shinyanga Region with a prevalence of HIV above the national average, which may not be generalizable to other study regions with lower HIV prevalence in Tanzania. "

OTHER INFORMATION

23) Registration number and name of trial registry

Does your paper address CONSORT subitem 23? *

Copy and paste relevant sections from the manuscript (include quotes in quotation marks "like this" to indicate direct quotes from your manuscript), or elaborate on this item by providing additional information not in the ms, or briefly explain why the item is not applicable/relevant for your study

"The trial is registered at ClinicalTrials.gov, number NCT03597243"

24) Where the full trial protocol can be accessed, if available

Does your paper address CONSORT subitem 24? *

Cite a Multimedia Appendix, other reference, or copy and paste relevant sections from the manuscript (include quotes in quotation marks "like this" to indicate direct quotes from your manuscript), or elaborate on this item by providing additional information not in the ms, or briefly explain why the item is not applicable/relevant for your study

The protocol is published in JMIR Research protocols

25) Sources of funding and other support (such as supply of drugs), role of funders

Does your paper address CONSORT subitem 25? *

Copy and paste relevant sections from the manuscript (include quotes in quotation marks "like this" to indicate direct quotes from your manuscript), or elaborate on this item by providing additional information not in the ms, or briefly explain why the item is not applicable/relevant for your study

"The study was funded by PEPFAR through USAID under Cooperative Agreement Number AID-621-A-15-00003"

X27) Conflicts of Interest (not a CONSORT item)

X27-i) State the relation of the study team towards the system being evaluated

In addition to the usual declaration of interests (financial or otherwise), also state the relation of the study team towards the system being evaluated, i.e., state if the authors/evaluators are distinct from or identical with the developers/sponsors of the intervention.

	1	2	3	4	5	
subitem not at all important	○	○	○	◉	○	essential

Clear selection

Does your paper address subitem X27-i?

Copy and paste relevant sections from the manuscript (include quotes in quotation marks "like this" to indicate direct quotes from your manuscript), or elaborate on this item by providing additional information not in the ms, or briefly explain why the item is not applicable/relevant for your study

The authors declared no competing interests

About the CONSORT EHEALTH checklist

As a result of using this checklist, did you make changes in your manuscript? *

- ○ yes, major changes
- ○ yes, minor changes
- ◉ no

What were the most important changes you made as a result of using this checklist?

I made no changes to the manuscript

How much time did you spend on going through the checklist INCLUDING making changes in your manuscript *

I made no changes to the manuscript

As a result of using this checklist, do you think your manuscript has improved? *

- ○ yes
- ○ no
- ◉ Other: I made no changes to the manuscript

Would you like to become involved in the CONSORT EHEALTH group?

This would involve for example becoming involved in participating in a workshop and writing an "Explanation and Elaboration" document

- ○ yes
- ◉ no
- ○ Other:

Clear selection

Any other comments or questions on CONSORT EHEALTH

Your answer

STOP - Save this form as PDF before you click submit

To generate a record that you filled in this form, we recommend to generate a PDF of this page (on a Mac, simply select "print" and then select "print as PDF") before you submit it.

When you submit your (revised) paper to JMIR, please upload the PDF as supplementary file.

Don't worry if some text in the textboxes is cut off, as we still have the complete information in our database. Thank you!

Final step: Click submit !

Click submit so we have your answers in our database!

Submit

Clear form

Never submit passwords through Google Forms.

This content is neither created nor endorsed by Google. Report Abuse - Terms of Service - Privacy Policy

Google Forms
